# Contributions of the Dachsous intracellular domain to Dachsous-Fat signaling

**DOI:** 10.1242/dev.202919

**Published:** 2024-11-29

**Authors:** Bipin Kumar Tripathi, Kenneth D. Irvine

**Affiliations:** Waksman Institute and Department of Molecular Biology and Biochemistry, Rutgers University, Piscataway, NJ 08854, USA

**Keywords:** Dachsous, DCHS1, Fat, Hippo, Planar cell polarity, Cross-vein

## Abstract

The protocadherins Fat and Dachsous regulate organ growth, shape, patterning, and planar cell polarity. Although Dachsous and Fat have been described as ligand and receptor, respectively, in a signal transduction pathway, there is also evidence for bidirectional signaling. Here, we assess signaling downstream of Dachsous through analysis of its intracellular domain. Genomic deletions of conserved sequences within *dachsous* identified regions of the intracellular domain that contribute to Dachsous activity. Deletion of the A motif increased Dachsous protein levels and decreased wing size. Deletion of the D motif decreased Dachsous levels at cell membranes, increased wing size, and disrupted wing, leg and hindgut patterning and planar cell polarity. Co-immunoprecipitation experiments established that the D motif is necessary and sufficient for association of Dachsous with key partners, including Lowfat, Dachs, Spiny-legs, Fat and MyoID. Subdivision of the D motif identified distinct regions that preferentially contribute to different Dachsous activities. Our results identify motifs that are essential for Dachsous function and are consistent with the hypothesis that the key function of Dachsous is regulation of Fat.

## Teaser

**Summary:** Protein binding and genetic studies identify and characterize structural and functional motifs within the intracellular domain of *Drosophila* Dachsous.

## INTRODUCTION

Dachsous-Fat signaling polarizes cells to regulate tissue polarity, growth, and patterning (reviewed by Fulford and McNeill, 2020; Gridnev and Misra, 2022; Strutt and Strutt, 2021). It is initiated by Dachsous (Ds) and Fat, two large cadherin proteins that bind each other through their extracellular domains (Clark et al., 1995; Mahoney et al., 1991; Matakatsu and Blair, 2004; Simon et al., 2010). Ds and Fat function in multiple organs throughout *Drosophila* development (reviewed by Fulford and McNeill, 2020; Gridnev and Misra, 2022; Strutt and Strutt, 2021). They are conserved in vertebrates, where they also function in multiple organs and have been linked to congenital diseases (Alders et al., 2014; Cappello et al., 2013; Durst et al., 2015; Mao et al., 2011; Saburi et al., 2008; Zakaria et al., 2014). The *Drosophila* wing has been a key organ used to dissect Ds-Fat signaling. *ds* mutants have enlarged, rounder wings, abnormalities in wing patterning, and abnormal wing hair planar cell polarity (PCP) (Adler et al., 1998; Baena-López et al., 2005; Clark et al., 1995). These phenotypes are shared by *fat* loss-of-function alleles or RNAi knockdown, but *ds* phenotypes are generally weaker than *fat* phenotypes (Bryant et al., 1988; Clark et al., 1995; Matakatsu and Blair, 2006). Binding between Ds and Fat is modulated by Four-jointed (Fj), a Golgi-localized kinase that phosphorylates their cadherin domains (Brittle et al., 2010; Ishikawa et al., 2008; Simon et al., 2010; Strutt and Strutt, 2002). Ds, Fj, and in some cases Fat are expressed in gradients across tissues where they function, and their differential expression and binding interactions leads to polarized membrane localization of Ds and Fat (Ambegaonkar et al., 2012; Brittle et al., 2012; Cho and Irvine, 2004; Clark et al., 1995; Ma et al., 2003; Matakatsu and Blair, 2004; Strutt and Strutt, 2002; Villano and Katz, 1995).

Ds and Fat regulate transcription through the Hippo pathway, and in this context Ds and Fat have been described as ligand and receptor, respectively (Bennett and Harvey, 2006; Cho et al., 2006; Mao et al., 2006; Matakatsu and Blair, 2006; Rogulja et al., 2008; Silva et al., 2006; Willecke et al., 2006, 2008). However, other observations suggest that Ds-Fat signaling should be considered as bidirectional, implying that they function as both ligand and receptor for each other, and PCP signaling is inherently bidirectional (Casal et al., 2006; Degoutin et al., 2013; Matakatsu and Blair, 2012; Willecke et al., 2008; Zecca and Struhl, 2010). Moreover, the observation that elimination of both Ds and Fat results in stronger phenotypes than elimination of Fat alone implies that Ds has functions beyond regulation of Fat (Matakatsu and Blair, 2006). Extensive studies of the Fat intracellular domain (ICD) have provided insights into how it mediates downstream signal transduction and supported its classification as a signal-transducing receptor (Fulford et al., 2023; Matakatsu and Blair, 2012; Pan et al., 2013; Zhao et al., 2013). However, comparable studies have not previously been described for Ds.

Several proteins that act within Ds-Fat signaling have been identified. One factor highly conserved in vertebrates is Lowfat (Lft), which maintains normal levels of Fat and Ds in the developing *Drosophila* wing, and which can physically associate with their cytoplasmic domains (Mao et al., 2009). A key downstream factor mediating the influence of Ds-Fat on both Hippo and PCP pathways in *Drosophila* is the atypical myosin Dachs (Ambegaonkar and Irvine, 2015; Ayukawa et al., 2014; Bosveld et al., 2012; Cho et al., 2006; Cho and Irvine, 2004; Mao et al., 2006). Ds-Fat signaling regulates the levels of Dachs membrane localization to modulate Hippo signaling, and the polarity of Dachs membrane localization to modulate PCP. In the developing wing imaginal disc, Dachs is localized to the distal sides of cells, where it often colocalizes with Ds (Ambegaonkar et al., 2012; Brittle et al., 2012; Mao et al., 2006). Dachs is removed from the proximal sides of cells in Fat-dependent process, and the significance of Ds-Dachs colocalization, and of the ability of Ds and Dachs to physically associate, has remained unclear. Dachs, together with the co-dependent factor Dlish (also known as Vamana), regulate Hippo signaling through effects on the Hippo pathway kinase Warts, and the upstream regulator Expanded (Cho et al., 2006; Feng and Irvine, 2007; Misra and Irvine, 2016; Vrabioiu and Struhl, 2015; Wang et al., 2019; Zhang et al., 2016). Ds-Fat polarize cells in part by regulating the Fz, or core, PCP pathway, and in part independently of Fz PCP signaling (reviewed by Strutt and Strutt, 2021). Crosstalk with Fz-PCP signaling is mediated through Spiny-legs (Sple), which is an isoform of Prickle (Pk) (Ambegaonkar and Irvine, 2015; Ayukawa et al., 2014; Gubb et al., 1999; Merkel et al., 2014; Olofsson et al., 2014). Dachs and Ds can each physically associate with Sple and contribute to polarized Sple localization.

Here, we use a structure-function approach to investigate potential signal transduction downstream of Ds. Ds is a 379 kD protein including a large extracellular domain with 27 cadherin repeats and a small (436 aa) intracellular domain (Clark et al., 1995). We identified and deleted six different conserved motifs within the Ds-ICD. Phenotypic analysis identified contributions of these motifs to Ds activity. Part of this can be ascribed to effects on Ds protein levels and distribution, as deletion of the A motif increases Ds protein levels, whereas deletion of the D motif decreases Ds protein at cell membranes. The change in Ds protein in the absence of the D motif can be explained by our discovery that it associates with and is required for regulation of Ds by Lft. The D motif also associates with other factors important to Ds-Fat signaling, including Dachs, Sple, Fat and MyoID (Myo31DF), and by making smaller deletions of the D motif the effects of Lft could be partially separated from other requirements for this motif.

## RESULTS

### Identification and deletion of conserved sequence motifs in the Ds-ICD

**Fig. 1.**
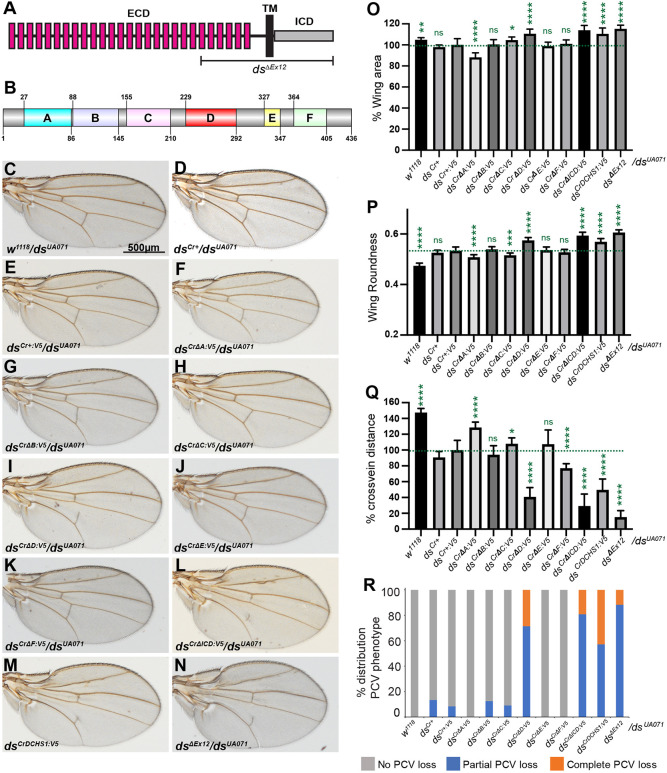
**Adult wing phenotypes of Ds-ICD motif deletions.** (A) Schematic of Ds, depicting cadherin domains (magenta rectangles) in the extracellular domain (ECD), transmembrane domain (TM), and intracellular domain (ICD). The region affected by exon 12 deletion is indicated (*ds*^Δ*Ex12*^). (B) Schematic of the location of conserved sequence motifs within the ICD. Amino acid numbers are indicated. (C-N) Male wings from *w^1118^/ds^UA071^* (*n*=26; C), *ds^Cr+^/ds^UA071^* (*n*=15; D), *ds^Cr+:V5^/ds^UA071^* (*n*=20; E), *ds^Cr^*^Δ*A:V5*^*/ds^UA071^* (*n*=18; F), *ds^Cr^*^Δ*B:V5*^*/ds^UA071^* (*n*=24; G), *ds^Cr^*^Δ*C:V5*^*/ds^UA071^* (*n*=22; H), *ds^Cr^*^Δ*D:V5*^*/ds^UA071^* (*n*=19; I), *ds^Cr^*^Δ*E:V5*^*/ds^UA071^* (*n*=18; J), *ds^Cr^*^Δ*F:V5*^*/ds^UA071^* (*n*=23; K), *ds^Cr^*^Δ*ICD:V5*^*/ds^UA071^* (*n*=16; L), *ds^CrDCHS1:V5^/ds^UA071^* (*n*=14; M), and *ds*^Δ*Ex12*^*/ds^UA071^* (*n*=30; N). Scale bar: 500 µm. (O-R) Histograms illustrating relative wing area (compared to that in *ds^Cr+:V5^/ds^UA071^*) (O), roundness (P), cross-vein distance (Q), and PCV loss (R) in the indicated genotypes. Error bars indicate mean±s.d., the significance of differences relative to *ds^Cr+^/ds^UA071^* is indicated by green asterisks and were calculated using one-way ANOVA on measurements from the number of wings indicated above. ns, not significant.

We assessed evolutionary conservation to guide identification of functionally important regions of the Ds-ICD. Conserved amino acids were clustered in six different regions (A to F), extending from near the transmembrane domain to the C terminus (Fig. 1B, Fig. S1). Four of these motifs (B, D, E, and F) are conserved from insects to vertebrates, whereas two (A and C) are only conserved within insects.

The ICD, transmembrane domain, and part of the extracellular domain are encoded by a single exon (exon 12) (Fig. 1A, Fig. S2A). To assess the functional significance of conserved motifs, we used recombination-mediated cassette exchange (RMCE) (Zhang et al., 2014), which enables efficient replacement through site-specific recombination of sequences flanked by attP sites. CRISPR-Cas9-mediated recombination was used to replace exon 12 with an RMCE cassette including an RFP gene flanked by attP sites in the upstream intron and 3′ UTR (Fig. S2A). This created a new mutant allele, *ds*^Δ*Ex12*^, which behaves as a strong *ds* allele with characteristic phenotypes, including wing overgrowth, rounder wings, reduced cross-vein spacing, abnormal hair polarity, and partial loss of the posterior cross-vein (PCV) (Fig. S2). The RMCE cassette was then replaced by site-specific recombination with either wild-type *ds* sequences, or with *ds* sequences in which one of the motifs A-F were deleted. We also created alleles in which the entire Ds-ICD was deleted (*ds^Cr^*^Δ*ICD:V5*^), and in which the ICD was replaced by the ICD from human DCHS1 (*ds^CrDCHS1:V5^*). We also included a C-terminal V5 tag so that the levels and distribution of the encoded Ds proteins could be examined.

### Identification of conserved motifs that contribute to Ds function

**Fig. 2.**
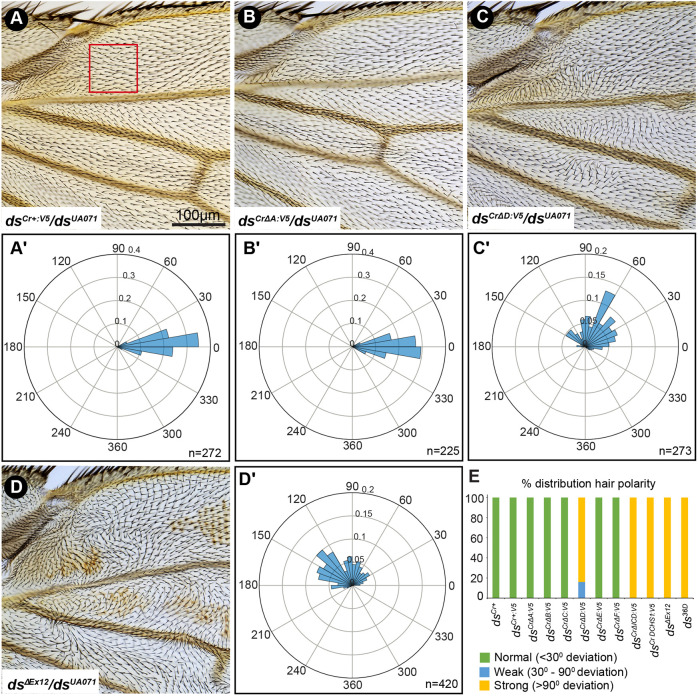
**Wing hair PCP phenotypes of Ds-ICD motif deletions.** (A-D) Male proximal anterior wings from *ds^Cr+:V5^/ds^UA071^* (A), *ds^Cr^*^Δ*A:V5*^*/ds^UA071^* (B), *ds^Cr^*^Δ*D:V5*^*/ds^UA071^* (C), and *ds*^Δ*Ex12*^*/ds^UA071^* (D). Scale bar: 100 µm. (A′-D′) Rose plots showing quantification of hair polarity from the wing region enclosed by the red box in A, from *ds^Cr+:V5^/ds^UA071^* (A′), *ds^Cr^*^Δ*A:V5*^*/ds^UA071^* (B′), *ds^Cr^*^Δ*D:V5*^*/ds^UA071^* (C′), and *ds*^Δ*Ex12*^*/ds^UA071^* (D′). Distal is to the right for each plot, and the number of hairs scored is indicated at the bottom right. (E) Histogram showing the distribution of proximal wing hair PCP in different alleles transheterozygous over *ds^UA071^* based on analysis of 14-30 wings per genotype (as listed in Fig. 1 legend). Wings were scored as normal if hairs deviated less than 30° from normal, weak if at least 10% of hairs deviated 30-90° from normal, and strong if at least 10% of hairs deviated >90° from normal.

To assess each *ds* allele, we examined them in transheterozygous combinations over the strong *ds* allele *ds^UA071^*. Unexpectedly, our allele in which the RMCE cassette was replaced by wild-type *ds* sequences, *ds^Cr+:V5^*, behaved as a weak *ds* allele. *ds^Cr+:V5^* wings were slightly smaller and rounder than wild-type (*w^1118^*) control wings (Fig. 1C,E,O,P, Fig. S2P-S), and the distance between cross-veins was reduced (Fig. 1Q, Fig. S2T). Wing hair polarity was, however, normal (Fig. 2A,E). The influence of *ds* on wing size is complex, as wings in animals with strong *ds* alleles are larger than those of wild type, whereas wings in animals with weak *ds* alleles are slightly smaller than those of wild type (Clark et al., 1995).

To investigate whether the V5 tag was interfering with Ds activity, we created an additional RMCE-mediated wild-type replacement allele without the tag (*ds^Cr+^*). However, comparison of *ds^Cr+^* and *ds^Cr+:V5^* revealed that they have similar phenotypes, including mild reductions in wing growth and cross-vein spacing, together with slightly increased wing roundness (Fig. 1D,O-R). DNA sequencing including all of exon 12 and an additional 1 kb upstream and downstream did not reveal any alterations other than the attP sequences in non-coding regions used to mediate RMCE. Thus, we conclude that the V5 tag does not affect Ds protein, but the inserted attP sequences may result in mild alterations of *ds* expression. As our goal was to compare the activities of different proteins, we continued with analysis of the different deletion alleles we had created, using *ds^Cr+:V5^* as our baseline for comparison to wild-type protein.

Three of the six alleles with deletions of conserved sequences exhibit altered wing sizes compared to *ds^Cr+:V5^*. *ds^Cr^*^Δ*A:V5*^ wings were on average 12% smaller than control wings (Fig. 1F,O), whereas *ds^Cr^*^Δ*C:V5*^ and *ds^Cr^*^Δ*D:V5*^ wings were on average 5% and 11%, respectively, larger than control wings (Fig. 1H,I,O). Conversely, wings in *ds^Cr^*^Δ*B:V5*^, *ds^Cr^*^Δ*E:V5*^ or *ds^Cr^*^Δ*F:V5*^ flies did not differ significantly in size from controls (Fig. 1G,J,K,O). In flies with deletion of the entire ICD, *ds^Cr^*^Δ*ICD:V5*^, wings were 14% larger than those of the *ds^Cr+:V5^* control (Fig. 1L,O), similar to the 16% increase in wing size in *ds*^Δ*Ex12*^ flies (Fig. 1N,O). In flies expressing *ds* with the human DCHS1 ICD, wings were 10% larger than those of controls (Fig. 1M,O).

The wing normally has an elongated shape, and both weak and strong *ds* alleles result in rounder wings (Clark et al., 1995). Wing roundness, measured as 4×[(area)/π×(major axis)^2^]), was increased in *ds^Cr^*^Δ*D:V5*^ wings compared to *ds^Cr+:V5^* controls, but was slightly reduced in *ds^Cr^*^Δ*A:V5*^ and *ds^Cr^*^Δ*C:V5*^ wings (Fig. 1P). Wing shape in *ds^Cr^*^Δ*B:V5*^, *ds^Cr^*^Δ*E:V5*^ and *ds^Cr^*^Δ*F:V5*^ did not differ significantly from controls. *ds*^Δ*Ex12*^, *ds^Cr^*^Δ*ICD:V5*^ and *ds^CrDCHS1:V5^* wings were rounder than control wings (Fig. 1P).

Reduced distance between anterior and posterior cross-veins is a characteristic feature of mutations in genes in the Ds-Fat pathway, including *ds*. Amongst the motif deletion alleles that we created, deletion of the D motif, in *ds^Cr^*^Δ*D:V5*^, had the strongest effect, with cross-vein spacing reduced by 59% compared to controls (Fig. 1I,Q). Cross-vein spacing was also reduced by 23% in *ds^Cr^*^Δ*F:V5*^ (Fig. 1K,Q). Cross-vein spacing was increased compared to controls by deletion of the A motif (by 28%) or deletion of the C motif (by 8%) (Fig. 1F,H,Q). No significant difference in cross-vein spacing was observed in *ds^Cr^*^Δ*B:V5*^ or *ds^Cr^*^Δ*E:V5*^ wings (Fig. 1G,J,Q). Deletion of the entire ICD resulted in a 71% reduction in cross-vein distance compared to controls, and cross-vein distance was reduced by 85% in *ds*^Δ*Ex12*^ (Fig. 1L,N,Q). In *ds^CrDCHS1:V5^*, cross-vein spacing was reduced by 50% (Fig. 1M,Q).

*ds* mutant alleles also often exhibited some loss of the PCV, which we categorized as: no PCV loss, partial PCV loss, or complete PCV loss (Fig. 1R). In *ds^Cr+:V5^*, 92% had no PCV loss, and 8% had partial PCV loss. Wings from *ds^Cr^*^Δ*B:V5*^ and *ds^Cr^*^Δ*C:V5*^ had PCV loss phenotypes similar to controls (88% and 91% with no PCV loss, respectively). All *ds^Cr^*^Δ*A:V5*^; *ds^Cr^*^Δ*E:V5*^ and *ds^Cr^*^Δ*F:V5*^ wings had no PCV loss. Conversely, PCV loss was increased in *ds^Cr^*^Δ*D:V5*^, with 29% having complete PCV loss and the remaining 71% with partial PCV loss. Strong PCV loss was also observed in *ds^Cr^*^Δ*ICD:V5*^, *ds^CrDCHS1:V5^*, and *ds*^Δ*Ex12*^.

Wing hairs are normally oriented from proximal to distal, but hair polarity is disrupted in PCP mutants. Ds-Fat pathway mutants have their strongest effects in proximal regions of the wing, and in wings from *ds* mutants misoriented hairs and swirling patterns were evident in the anterior proximal wing (Fig. 2D). Control flies expressing wild-type Ds protein (*ds^Cr+:V5^*) or flies expressing *ds^Cr^*^Δ*A:V5*^ did not exhibit wing hair PCP phenotypes (Fig. 2A-B′,E). Flies expressing *ds*^Δ*Ex12*^ *ds^Cr^*^Δ*ICD:V5*^, or *ds^CrDCHS1:V5^* had strong hair polarity defects (hairs deviated more than 90° from normal orientation) in all of the wings examined (Fig. 2D-E, Fig. S3F,G). *ds^CrΔD:V5^* also had consistent hair polarity defects, with 84% of wings examined showing strong polarity defects and 16% of wings showing weak defects (hairs deviated between 30° and 90° from normal orientation) (Fig. 2C,C′,E). Wings from *ds^Cr^*^Δ*B:V5*^; *ds^Cr^*^Δ*C:V5*^; *ds^Cr^*^Δ*E:V5*^ and *ds^Cr^*^Δ*F:V5*^ flies had no evident wing hair polarity phenotypes (Fig. 2E, Fig. S3B-E).

In *ds* mutants, legs are shorter and wider than wild-type legs and the number of tarsal segments is reduced (Fig. S3S,T) (Clark et al., 1995). A reduced number of tarsal segments was observed in *ds^Cr^*^Δ*D:V5*^ flies, as well as *ds^Cr^*^Δ*ICD:V5*^, *ds^CrDCHS1:V5^*, and *ds^ΔEx12^* flies (Fig. S3M,P,Q,R). Conversely, *ds^Cr^*^Δ*A:V5*^, *ds^Cr^*^Δ*B:V5*^, *ds^Cr^*^Δ*C:V5*^, *ds^Cr^*^Δ*E*^*^:V5^* and *ds^Cr^*^Δ*F:V5*^ lines all had five tarsal segments, as observed in control flies *ds^Cr+:V5^* and *w^1118^* (Fig. S3I-L,N,O).

In summary (Table S1), deletion of the D motif results in phenotypes consistent with reduced Ds activity across a range of activities whereas deletion of the A motif, and to a lesser extent the C motif, result in phenotypes consistent with increased Ds activity. Deletion of the B, E, or F motifs has essentially no effect.

### Influence of Ds-ICD deletions on Ds protein localization and levels

**Fig. 3.**
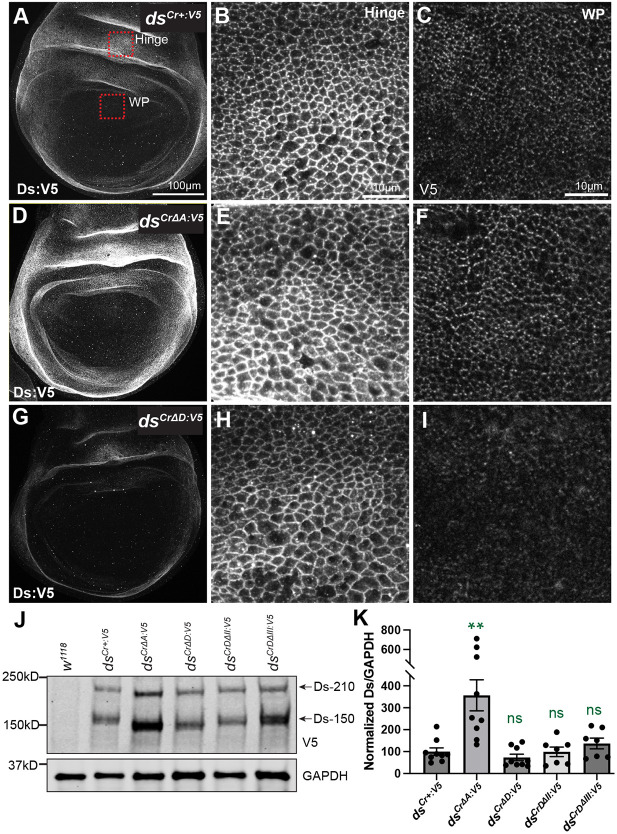
**Localization and levels of Ds protein.** Third-instar wing discs expressing Ds:V5 protein from homozygous *ds^Cr+:V5^* (A-C), *ds^Cr^*^Δ*A:V5*^ (D-F), and *ds^Cr^*^Δ*D:V5*^ (G-I). Red boxes in A show approximate locations in hinge and wing pouch (WP) corresponding to the higher magnification images on the right. (A,D,G) Wing pouch and hinge regions. Scale bar: 100 µm. (B,E,H) Part of the wing hinge region from homozygous *ds^Cr+:V5^* (B), *ds^Cr^*^Δ*A:V5*^ (E), and *ds^Cr^*^Δ*D:V5*^ (H). Scale bar: 10 µm. (C,F,I) Proximal wing pouch from homozygous *ds^Cr+:V5^* (C), *ds^Cr^*^Δ*A:V5*^ (F), and *ds^Cr^*^Δ*D:V5*^ (I). Scale bar: 10 µm. (J) Western blot on lysates of third instar wing discs showing relative levels of Ds:V5 in the indicated genotypes. Ds is processed at two alternative sites in its extracellular domain, resulting in the Ds-210 and Ds-150 bands detected here (Ambegaonkar et al., 2012). Both Ds-210 and Ds-150 intensities are included in the quantification. (K) Histogram showing quantification of western blots, based on nine replicates for *ds^Cr+:V5^*, *ds^Cr^*^Δ*A:V5*^ and *ds^Cr^*^Δ*D:V5*^, and seven replicates for *ds^CrD^*^Δ*II:V5*^and *ds^CrD^*^Δ*III:V5*^. *ds^CrD^*^Δ*II:V5*^and *ds^CrD^*^Δ*III:V5*^ are described in the section ‘Subdivision of the D region identifies multiple, partially separable activities’. Statistical significance of differences compared to *ds^Cr+:V5^* is in green. ns, not significant.

To investigate the basis for the influence of these alleles on Ds activity, we first examined the localization and levels of the Ds protein expressed by them. Examination of Ds:V5 protein expressed by *ds^Cr+:V5^* revealed a distribution similar to that previously described for Ds, including a proximal-to-distal gradient with relatively high levels in the wing hinge, low levels in the proximal wing pouch, and barely detectable levels in the distal wing pouch (Fig. 3A). As for endogenous Ds, Ds:V5 protein was localized near apical cell junctions, and at higher magnification often appeared somewhat punctate (Fig. 3B,C).

To compare Ds expression in different alleles, we performed staining and imaging in parallel using identical conditions. All of the alleles express Ds:V5 in a proximal-to-distal gradient (Fig. 3D,G, Fig. S4). For *ds^Cr^*^Δ*B:V5*^; *ds^Cr^*^Δ*C:V5*^; *ds^Cr^*^Δ*E:V5*^ and *ds^Cr^*^Δ*F:V5*^, each of which provided essentially normal Ds activity, Ds:V5 protein expression appeared to be similar to Ds:V5 expressed by *ds^Cr+:V5^* (Fig. S4A-L). For Ds:V5 expressed by *ds^Cr^*^Δ*A:V5*^, which provides enhanced Ds activity, protein levels appeared increased relative to *ds^Cr+:V5^* (Fig. 3D). For Ds:V5 expressed from *ds^Cr^*^Δ*D:V5*^, which provides decreased Ds activity, protein levels appear reduced relative to *ds^Cr+:V5^* (Fig. 3G). The increases and decreases in Ds:V5 staining were observed across different regions of the wing disc, including the pouch and hinge (Fig. 3D-I). Ds:V5 staining appeared much reduced and poorly localized in *ds^Cr^*^Δ*ICD:V5*^ wing discs (Fig. S4M-O), which suggests that the ICD is important for normal localization and levels of Ds. Examination of wing discs from *ds^CrDCHS1:V5^* revealed that the Ds-DCHS1 hybrid protein is not properly localized, as Ds:DCHS1:V5 accumulates inside cells, possibly due to mis-folding (Fig. S4P-R).

Western blot analysis on alleles for which immunostaining identified differences in expression confirmed that deleting the A motif leads to increased levels of Ds protein (Fig. 3J,K). A slight decrease in Ds levels was detected by western blotting when the D motif was deleted, but it was not statistically significant (Fig. 3J,K). Thus, we infer that the reduced cell membrane staining detected in *ds^Cr^*^Δ*D:V5*^ wing discs primarily reflects mis-localization rather than decreased protein levels.

To compare the Ds expressed by our *ds:V5* alleles with endogenous Ds, we generated mitotic clones in wing discs heterozygous for *ds^Cr+:V5^*, *ds^Cr^*^Δ*A:V5*^, and *ds^Cr^*^Δ*D:V5*^, and stained using anti-Ds antisera. Ds staining in clones of homozygous *ds^Cr+:V5^* cells was slightly weaker than that of neighboring heterozygous cells (Fig. S5A), which could explain the mild phenotype induced by this allele. Ds staining in clones of homozygous *ds^Cr^*^Δ*D:V5*^ cells was even weaker compared to neighboring heterozygous cells, whereas staining in clones of homozygous *ds^Cr^*^Δ*A:V5*^ cells was comparable to that of neighboring heterozygous cells (Fig. S5B,C); these observations are consistent with our analysis of V5 staining.

### The D motif of Ds is required for regulation by Lft

**Fig. 4.**
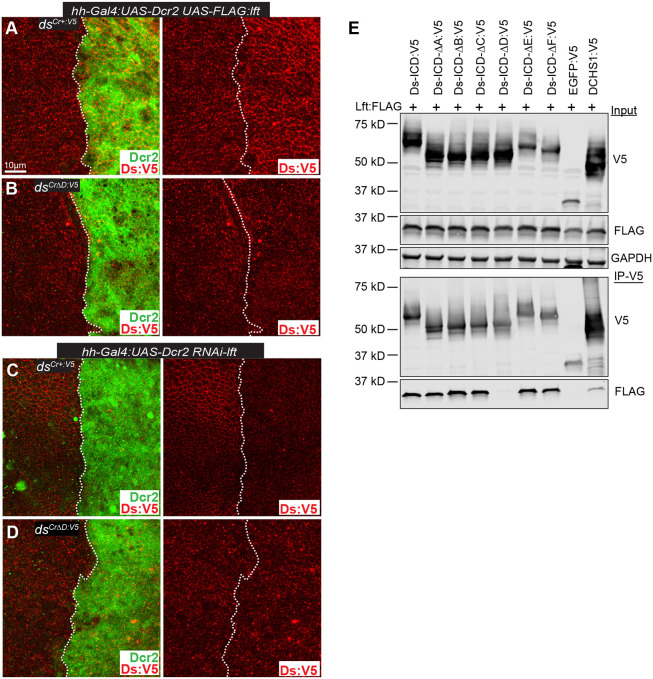
**The D motif mediates regulation of Ds by Lft.** (A,B) Wing discs expressing *hh*-*Gal4*-driven *UAS*-*FLAG*:*lft* in *ds^Cr+:V5^* (A) or *ds^Cr^*^Δ*D:V5*^ (B) with UAS-Dcr2 marking posterior (green), stained for Ds:V5 (red). (C,D) Wing discs expressing *hh*-*Gal4*-driven *UAS-RNAi-lft* in *ds^Cr+:V5^* (C) or *ds^Cr^*^Δ*D:V5*^ (D) with UAS-Dcr2 marking posterior (green), stained for Ds:V5 (red). Dotted lines in A-D delineate the anterior-posterior boundary, with the region to the right of the dotted line being the posterior compartment. (E) Western blot showing results of co-immunoprecipitation experiments between Ds:V5 and FLAG:Lft expressed in S2 cells. Top three panels show blots of cell lysates expressing the indicated proteins, using the antibodies indicated on the right. GAPDH is a control for loading and transfer. Bottom two panels show blots on proteins immunoprecipitated with anti-V5 beads and detected with V5 or FLAG antibodies, as indicated.

The decreased levels of Ds at cell membranes observed in *ds^Cr^*^Δ*D:V5*^ suggest that the D motif might be required for regulation of Ds by a factor acting through this motif. The *lft* gene acts post-transcriptionally to promote normal membrane levels of Fat and Ds in wing discs (Mao et al., 2009). To examine whether Lft regulation is mediated through the D motif, we compared Lft overexpression and knockdown in control *ds^Cr+:V5^* wing discs and *ds^Cr^*^Δ*D:V5*^ wing discs. In *ds^Cr^*^+*:*V5^, Lft over-expression in posterior compartments from a UAS transgene under *hh-Gal4* control increased visible levels of Ds:V5 in posterior cells compared to anterior cells (Fig. 4A), whereas depletion of Lft in posterior cells achieved using a *UAS-RNAi-lft* transgene decreased Ds:V5 compared to anterior cells (Fig. 4C). Conversely, in *ds^Cr^*^Δ*D:V5*^ discs, overexpression of Lft in posterior cells did not noticeably increase DsΔD:V5 staining compared to levels in anterior cells (Fig. 4B), and depletion of Lft did not visibly decrease DsΔD:V5 staining in posterior cells compared to anterior cells (Fig. 4D). These observations suggest that the reduced levels of Ds:V5 protein observed in *ds^Cr^*^Δ*D:V5*^ discs could be due to an inability to be positively regulated by Lft.

To investigate whether this reflects direct association with Lft through this motif, we mapped regions required for Lft binding. Earlier studies reported that Lft could associate with the Ds-ICD but did not identify where it binds (Mao et al., 2009). We expressed the ICDs of Ds motif deletion constructs in cultured *Drosophila* S2 cells, together with FLAG-tagged Lft. The intact Ds-ICD could co-immunoprecipitate Lft. Deletion of motifs A, B, C, E, or F did not affect the ability of Lft to co-immunoprecipitate with the Ds-ICD. Conversely, deletion of the D motif (Ds-ICD-ΔD:V5) resulted in loss of Lft–Ds-ICD co-precipitation (Fig. 4E). Thus, the D motif is uniquely required for association with Lft. Together with the insensitivity of the *ds^Cr^*^Δ*D:V5*^ mutant to altered expression of Lft, these results imply that the association of Lft with the D motif is required for Ds regulation.

The ability of Lft to associate with Fat and Ds is conserved by its mammalian homologs LIX1 and LIX1L (Mao et al., 2009), and we observed that deletion of the D motif also eliminated the binding of LIX1 and LIX1L to the Ds-ICD (Fig. S6). Conservation across species of the ability of Ds and Lft to bind to each is further supported by the observation that Lft was co-immunoprecipitated by the DCHS1 ICD (Fig. 4E).

### The D motif of Ds is required for Dachs regulation and binding

Fat promotes removal of Dachs from cell membranes, driving the changes in levels of membrane Dachs that influence Hippo signaling and the polarization of Dachs that influences PCP (reviewed by Fulford and McNeill, 2020; Gridnev and Misra, 2022; Strutt and Strutt, 2021). Dachs can colocalize in puncta with Ds and physically associate with the Ds-ICD (Ambegaonkar et al., 2012; Bosveld et al., 2012; Brittle et al., 2012), but the mechanistic significance of Ds-Dachs association to Dachs regulation remains unknown.

**Fig. 5.**
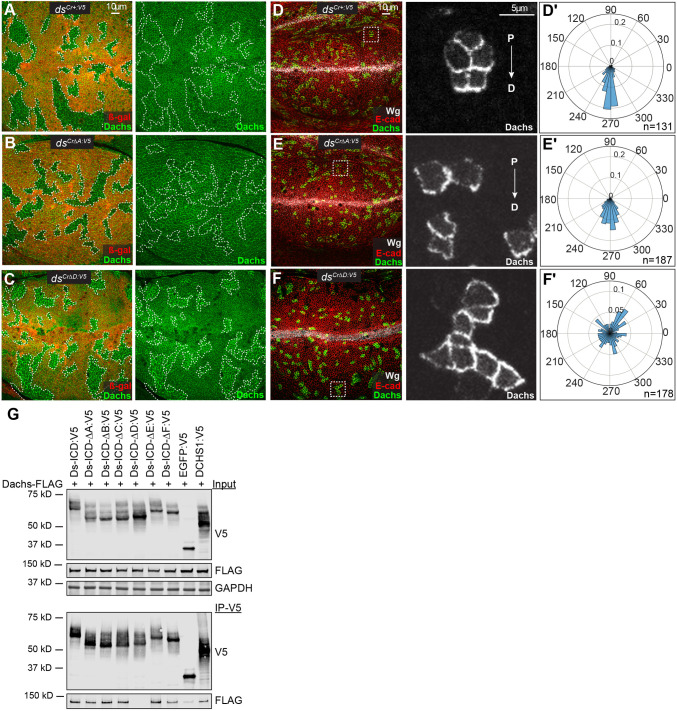
**The Ds-D motif is required for Dachs regulation and binding.** (A-C) Wing discs of *hs-Flp*; *ds^Cr+:V5^ FRT40A/arm-lacZ FRT40A; Dachs:GFP/+* (A), *hs-Flp*; *ds^Cr^*^Δ*A:V5*^ *FRT40A/arm-lacZ FRT40A; Dachs:GFP/+* (B), or *hs-Flp*; *ds^Cr^*^Δ*D:V5*^ *FRT40A/arm-lacZ FRT40A; Dachs:GFP/+* (C), with mitotic clones homozygous for *ds^Cr+:V5^*, *ds^Cr^*^Δ*A:V5*^, or *ds^Cr^*^Δ*D:V5*^, marked by loss of β-galactosidase (red), outlined with white dashes, to show effects on Dachs:GFP (green). (D-F) Wing discs of *hs-Flp*; *ds^Cr+:V5^/ds*^Δ*Ex12*^*; Act>stop>EGFP:Dachs/+* (D), *hs-Flp*; *ds^Cr^*^Δ*A:V5*^*/ds*^Δ*Ex12*^*; Act>stop>EGFP:Dachs/+* (E), or *hs-Flp*; *ds^Cr^*^Δ*D:V5*^*/ds*^Δ*Ex12*^*; Act>stop>EGFP:Dachs/+* (F) stained for Wg (white) and E-cadherin (Shotgun) (red) with clones expressing Dachs:GFP (green/white). Panels on the right show Dachs:GFP (white) in higher magnification images of the boxed regions. Proximal (P) to distal (D) orientation is indicated. (D′-F′) Rose plots showing quantification of Dachs polarity in Flp-out clones generated in the genotypes shown in D-F, respectively, based on *n*=131 cells from seven discs for (D′), *n*=187 cells from eight discs for (E′) and *n*=178 cells from six discs for (F′), and with distal at bottom. (G) Western blot showing results of co-immunoprecipitation experiments between Ds:V5 proteins and FLAG:Dachs transfected into S2 cells. Top three panels show blots on total cell lysates expressing the indicated proteins, using the antibodies indicated on the right. Bottom two panels show blots on proteins immunoprecipitated with anti-V5 beads and detected with V5 or FLAG antibodies.

To investigate this, we generated mitotic clones in wing discs for the Ds-ICD motif deletions that have significant phenotypes, and examined Dachs protein, using genomic GFP-labeled Dachs. Dachs was enriched at the subapical membrane in *ds^Cr^*^Δ*D:V5*^ mutant clones (Fig. 5C), whereas Dachs levels appeared to be unaffected in *ds^Cr^*^+*:V5*^ control clones (Fig. 5A). Mitotic clones of *ds^Cr^*^Δ*A:V5*^ also did not have evident effects on Dachs levels or localization (Fig. 5B).

To determine the influence of Ds-ICD motif deletions on Dachs polarization, we made small Flp-out clones expressing GFP-tagged Dachs, which facilitates visualization of polarization. A Flip-out cassette expressing Dachs:GFP with an intervening transcriptional stop cassette flanked by FRT sites was introduced into Ds-ICD motif deletion alleles. Heat-shock-induced Flipase expression led to Dachs:GFP-expressing clones. Most clones in *ds^Cr^*^+*:V5*^ wing discs exhibited normal, distally oriented Dachs polarity (Fig. 5D,D′). Dachs was also normally polarized in *ds^Cr^*^Δ*A:V5*^ wing discs (Fig. 5E,E′). Conversely, most Dachs:GFP clones in *ds^Cr^*^Δ*D:V5*^ mutant wing discs had mispolarized Dachs (Fig. 5F,F′). This mispolarization is consistent with the observation that PCP is abnormal in *ds^Cr^*^Δ*D:V5*^.

We also mapped regions of the Ds-ICD required for association with Dachs using a Flag-tagged Dachs construct and V5-tagged Ds-ICD constructs. Deletion of the A, B, C, E, or F motifs did not impair the ability to bind Dachs. Conversely, deletion of the D motif abolished detectable Dachs binding (Fig. 5G). We also found that V5-tagged DCHS1 can co-precipitate Dachs, consistent with the conclusion that Ds-Dachs association is mediated by a conserved motif.

### The D motif of Ds is required for Sple binding

The Sple isoform of the Pk-Sple locus links Ds-Fat PCP with Fz-PCP and can physically associate with Ds and Dachs and colocalize in puncta with Ds and Dachs (Ambegaonkar and Irvine, 2015; Ayukawa et al., 2014; Gubb et al., 1999; Merkel et al., 2014; Olofsson et al., 2014). We determined whether any conserved motifs are required for association with Sple using FLAG-tagged Sple-N-terminal region and V5-tagged Ds-ICD constructs. Deletion of the A, B, C, E, or F motifs did not impair the ability of the Ds-ICD to bind Sple-N. Conversely, deletion of the D motif eliminated detectable binding to Sple-N (Fig. S7), implying that Sple associates with Ds through the D motif of the ICD. This conclusion is consistent with the observation that *ds^Cr^*^Δ*D:V5*^ is the only one of the motif deletions that exhibited significant PCP phenotypes (Fig. 2C,E). However, as motif D also influences levels of Ds and localization of Dachs, the PCP phenotypes observed do not necessarily stem directly from impairment of association with Sple.

### The D motif of Ds is required for Fat-ICD binding

Recent research has revealed that, in addition to binding through their extracellular domains, Ds and Fat can also interact through their intracellular domains (Fulford et al., 2023). To identify Ds regions required for this, we conducted co-immunoprecipitation experiments in S2 cells, using V5-tagged Ds-ICD constructs and Alfa-tagged Fat-ICD. This revealed that deletion of the D motif specifically reduces Ds-ICD association with the Fat-ICD (Fig. S8).

### The D motif of Ds is sufficient for association with Lft, Dachs, and Sple

The observation that multiple binding partners of Ds each require the D motif for association with Ds raised concerns about the specificity of this requirement. For example, if deletion of the D motif results in misfolded protein, then the requirement might be indirect. We thus investigated whether the D motif is also sufficient for interaction with Lft, Dachs, and Sple. As the D motif is only 64 amino acids, this was achieved by creating and expressing a Ds-ICD D-motif:EGFP:V5 fusion protein, which was then used in co-immunoprecipitation experiments, alongside EGFP:V5 as a negative control. This revealed that Ds-D+EGFP:V5 could specifically co-immunoprecipitate Lft in S2 cells, and thus that the D motif is sufficient for Lft binding (Fig. S9A). Similarly, Ds-D+EGFP:V5, but not EGFP:V5 could co-immunoprecipitate Dachs and Sple (Fig. S9B,C).

### Subdivision of the D region identifies multiple, partially separable activities

**Fig. 6.**
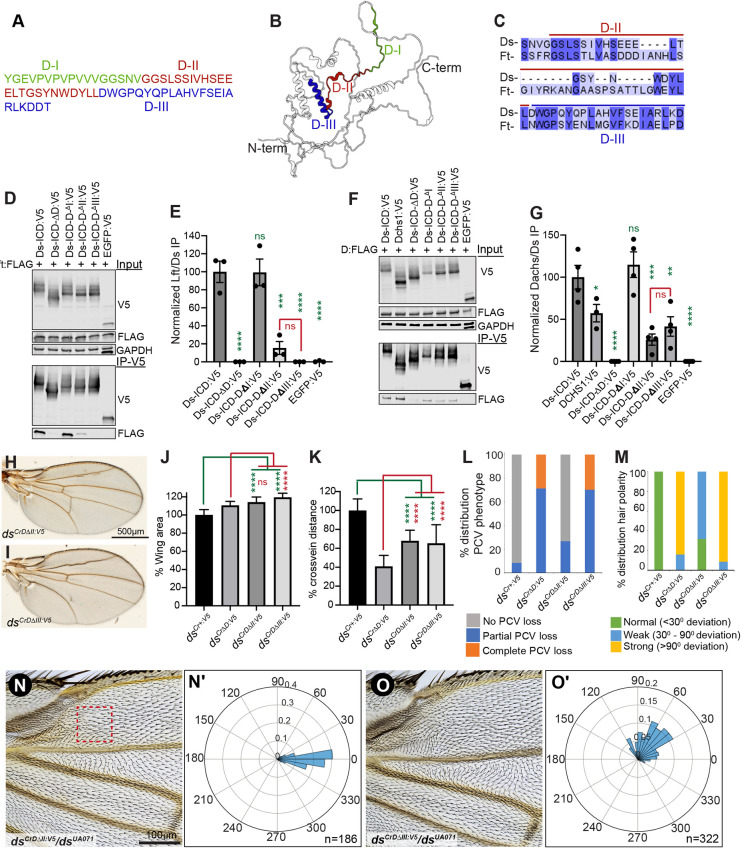
**Separating functions encoded by the Ds D region.** (A) Sequence of the D motif, with regions I, II, and III in green, red, and blue letters, respectively. (B) Predicted structure of the Ds-ICD, with regions, I, II and III indicated in colors as in A. (C) Comparison of a portion of the Fat-ICD and the Ds-D region (Clark et al., 1995; Mao et al., 2009). (D,F) Western blots showing results of co-immunoprecipitation experiments between V5-tagged Ds proteins and FLAG-tagged Lft (D), or Dachs (F), co-expressed in S2 cells. Top three panels show blots of cell lysates expressing the indicated proteins, detected using antibodies indicated on the right. Bottom two panels show blots on proteins immunoprecipitated with anti-V5 beads and detected with V5 or FLAG antibody. (E,G) Quantitation of relative co-precipitation of Lft (three replicates) or Dachs (four replicates) with the indicated Ds-ICD constructs. Significance of differences (by one-way ANOVA) in relative co-precipitation compared to Ds-ICD:V5 indicated in green, and comparison between DII and DIII deletions in red. (H,I) *ds^CrD^*^Δ*II:V5*^*/ds^UA071^* (H) or *ds^CrD^*^Δ*III:V5*^*/ds^UA071^* (I) male wings. (J-M) Histograms representing relative wing area (J), cross-vein distance (K), distribution of PCV loss (L) and distribution of PCP phenotypes (M) from the given *ds* alleles over *ds^UA071^*. Mean±s.d. shown from measurement of 24 (*ds^CrD^*^Δ*II:V5*^) or 25 (*ds^CrD^*^Δ*III:V5*^) wings. (N,O) Proximal anterior region of wings from *ds^CrD^*^Δ*II:V5*^*/ds^UA071^* (N) or *ds^CrD^*^Δ*III:V5*^*/ds^UA071^* (O)*.* Scale bar: 100 µm. (N′,O′) Rose plots showing quantification of hair polarity from the wing region boxed in N, from *ds^CrD^*^Δ*II:V5*^*/ds^UA071^* (N′) and *ds^CrD^*^Δ*III:V5*^*/ds^UA071^* (O′), with number of hairs measured indicated by *n*. ns, not significant.

We next investigated whether activities of the D motif could be separated. Structural prediction using AlphaFold (Jumper et al., 2021) suggested that the ICD is mostly disordered, but structured regions occurred in motifs B, C, and D (Fig. 6B). If Lft, Dachs, and Sple have differential binding regions, this might be revealed by smaller deletions. Using the predicted structure as a guide, we thus subdivided the D motif into three smaller regions, which we refer to as D-I, D-II, and D-III (Fig. 6A,B). We note that a region of the Fat-ICD with sequence similarity to the Ds-ICD overlaps the D motif (Clark et al., 1995; Mao et al., 2009), with maximum similarity in the D-II and D-III regions (Fig. 6C).

Constructs with smaller deletions within the D motif were expressed in S2 cells together with tagged Lft, Dachs, or Sple. Co-immunoprecipitation revealed that removing the D-I region does not affect binding of the Ds-ICD to Lft (Fig. 6D,E). However, deleting the D-II region reduced binding of Lft to 15% of Ds-ICD full-length levels (Fig. 6D,E), and deleting the D-III region eliminated binding of Lft to the Ds-ICD (Fig. 6D,E). Association of Dachs with the Ds-ICD was unaffected by deletion of the D-I region, but reduced to 26% of Ds-ICD full-length levels by deletion of the D-II region, and to 42% by deletion of the D-III region (Fig. 6F,G). Association of Sple with the Ds-ICD was reduced to 30% of Ds-ICD full-length levels by deletion of D-II and to 35% by deletion of D-III, although the reduction with D-III deletion was not statistically significant (Fig. S7C,D). Altogether, these results suggest that D-II and D-III, but not D-I, contribute to association of Lft, Dachs and Sple with Ds. The differences in relative strength of binding of Lft, Dachs or Sple to D-II versus D-III were not statistically significant (Fig. 6E,G, Fig. S7D).

Based on this, we generated flies with genomic deletions of the D-II or D-III regions, and compared their phenotypes to those of *ds^Cr^*^+*:V5*^ and *ds^Cr^*^Δ*D:V5*^. Deletion of the D-II region, *ds^CrD^*^Δ*II:V5*^, which reduces Dachs, Sple and Lft binding, led to an increase in wing size similar to that of *ds^Cr^*^Δ*D:V5*^ flies (Fig. 6H,J). Cross-vein spacing was decreased compared to *ds^Cr^*^+*:V5*^, but less so than in *ds^Cr^*^Δ*D:V5*^ flies (Fig. 6K). Loss of the PCV, and disruption of wing hair polarity was observed, but these phenotypes were substantially weaker than in *ds^Cr^*^Δ*D:V5*^ flies (Fig. 6L-O′). Thus, some functions of the Ds-ICD, related to its contributions to wing growth, are severely compromised by the D-II deletion, but other functions, including those related to PCP, are only partially compromised.

Deletion of the D-III region, *ds^CrD^*^Δ*III:V5*^, which eliminates Lft binding and reduces Dachs binding, led to an increase in wing size that was even greater than that observed in *ds^Cr^*^Δ*D:V5*^ flies (19% versus 11% larger than controls; Fig. 6I,J). Loss of the PCV and disruption of wing hair polarity phenotypes were also observed and appeared similar in strength to the complete deletion of the D motif (Fig. 6L-O′). D-III deletion wings also showed a decrease in cross-vein spacing (Fig. 6K), although this phenotype was milder than with full D-motif deletion. Overall though, removal of the D-III region resulted in phenotypes that were similar to those observed in *ds^Cr^*^Δ*D:V5*^.

**Fig. 7.**
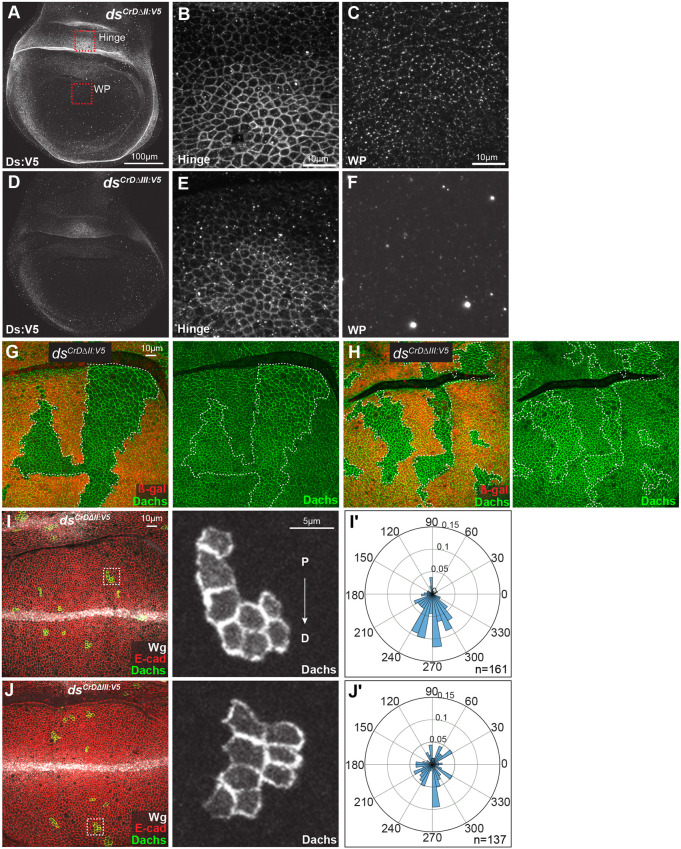
**Influence of D-II and D-III deletions on Ds and Dachs.** (A-F) Wing discs from homozygous *ds^CrD^*^Δ*II:V5*^ (A-C) or *ds^CrD^*^Δ*III:V5*^ (D-F) stained for Ds:V5. Red boxes in A show approximate locations in hinge and wing pouch (WP) corresponding to the higher magnification panels on the right. (A,D) Whole wing pouch and hinge regions. Scale bar: 100 µm. (B,C,E,F) Higher magnification images depicting part of the wing hinge (B,E) or proximal wing pouch (C,F). Scale bars: 10 µm. (G,H) Wing discs from *hs-Flp*; *ds^CrD^*^Δ*II:V5*^ *FRT40A/arm-lacZ FRT40A; Dachs:GFP/+* (G), or *hs-Flp*; *ds^CrD^*^Δ*III:V5*^ *FRT40A/arm-lacZ FRT40A; Dachs:GFP/+* (H), with clones homozygous for *ds^CrD^*^Δ*II:V5*^ (G) or *ds^CrD^*^Δ*III:V5*^ (H), marked by loss of β-galactosidase (red), outlined with white dashes, to show effects on Dachs:GFP (green). (I,J) Wing discs from *hs-Flp*; *ds^CrD^*^Δ*II:V5*^*/ds*^Δ*Ex12*^*; Act>stop>EGFP:Dachs/+* (I), or *hs-Flp*; *ds^CrD^*^Δ*III:V5*^*/ds*^Δ*Ex12*^*; Act>stop>EGFP:Dachs/+* (J), stained for expression of Wg (white) and E-cadherin (red) with clones expressing Dachs:GFP (green/white). Panels on the right show Dachs:GFP (white) in higher magnification images of the boxed regions. Proximal (P) to distal (D) orientation is indicated. (I′,J′) Rose plots showing quantification of Dachs polarity, with distal at bottom, in Flp-out clones from the genotypes shown in I (*n*=161 cells from nine discs) and J (*n*=137 cells from ten discs).

We also examined Ds protein levels and localization by immunostaining and western blotting. Ds protein localization in D-II deletion mutants appeared overall similar to that in *ds^Cr^*^+*:V5*^ control flies (Fig. 7A-C). Conversely, Ds protein localization in D-III deletion mutants showed reduced Ds localization to cell junctions, similar to that observed in *ds^Cr^*^Δ*D:V5*^ flies (Fig. 7D-F). Deleting the D-II or D-III motifs did not significantly alter Ds protein levels as assayed by western blotting (Fig. 3J,K).

We next examined Dachs levels and localization. Mitotic clones homozygous for *ds^CrD^*^Δ*II:V5*^ or *ds^CrD^*^Δ*III:V5*^ revealed that Dachs is elevated at the subapical membrane by both D-II and D-III motif deletions compared with Dachs in neighboring cells (Fig. 7G,H). This is consistent with the observation that these mutations are associated with increased wing growth, as membrane levels of Dachs correlate with its downregulation of Wts (Brittle et al., 2012; Mao et al., 2006; Rogulja et al., 2008; Vrabioiu and Struhl, 2015). Examination of Flp-out clones expressing Dachs:GFP revealed that Dachs polarity is often normal in flies with the D-II deletion but mostly mis-polarized in flies with the D-III deletion (Fig. 7I-J′). The distinct effects of these small deletions on Dachs polarity correlates with their distinct effects on wing hair PCP, cross-vein spacing, and PCV loss, which are strongly affected by D-III but only mildly affected by D-II. The stronger effects of the D-III deletion could be due to the reduced levels of Ds protein at membranes.

### Influence of Ds-ICD deletions on Hindgut looping and MyoID binding

**Fig. 8.**
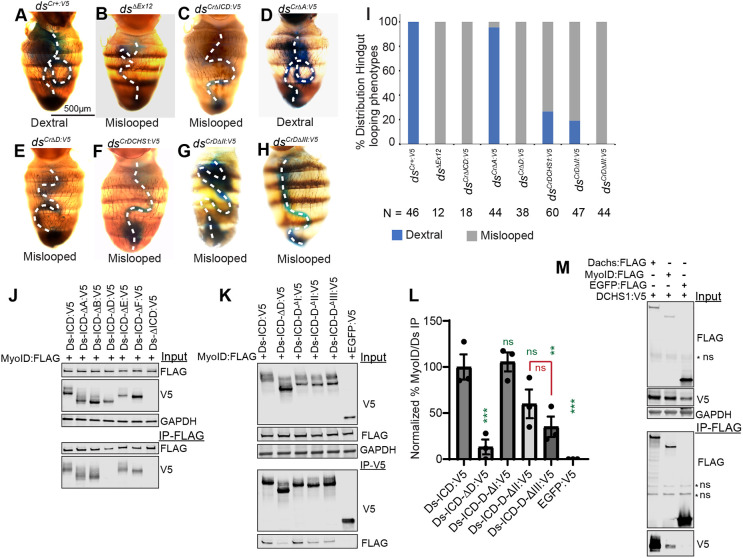
**The D motif is required for dextral hindgut looping and MyoID binding.** (A-H) Abdomens of flies fed dye to mark the hindgut. (A) *ds^Cr+:V5^/ds^UA071^* (control), (B) *ds^36D^/ds^UA071^*, (C) *ds^Cr^*^Δ*ICD:V5*^*/ds^UA071^*, (D) *ds^Cr^*^Δ*A:V5*^*/ds^UA071^,* (E) *ds^Cr^*^Δ*D:V5*^*/ds^UA071^*, (F) *ds^CrDCHS1:V5^/ds^UA071^*, (G) *ds^CrD^*^Δ*II:V5*^*/ds^UA071^*, (H) *ds^CrD^*^Δ*III:V5*^*/ds^UA071^.* Dashed lines indicate the path of the hindgut. (I) Histogram showing distribution of phenotypes, scored as normal dextral looping (blue), or mislooped (gray). *N*=number scored. (J,K) Western blots showing results of co-immunoprecipitation experiments between Ds:V5 proteins and MyoID:FLAG. Top three panels show blots on cell lysates expressing the indicated proteins, using the antibodies indicated on the right. Bottom two panels show blots on proteins immunoprecipitated with anti-FLAG beads (J) and anti-V5 beads (K) and detected with V5 or FLAG antibodies. (L) Quantitation of relative co-precipitation of MyoID with the indicated Ds-ICD:V5 constructs. Significance of differences (by one-way ANOVA) in relative co-precipitation compared to Ds-ICD:V5 indicated in green, and comparison between DII and DIII deletions in red. (M) Western blot showing results of co-immunoprecipitation experiments between V5-tagged DCHS1 ICD and the indicated FLAG-tagged proteins. Top three panels show blots on total cell lysates expressing the indicated proteins, using the antibodies indicated on the right. Bottom two panels show blots on proteins immunoprecipitated with anti-FLAG beads and detected with V5 or FLAG antibodies. Some non-specific (ns) bands are indicated.

Ds is also required for normal looping of the hindgut (González-Morales et al., 2015). In wild-type flies, the adult hindgut exhibits a clockwise coiling pattern (dextral looping), forming a single loop that is located on the right side of the abdomen when viewed dorsally. Flies mutant for the myosin family protein MyoID exhibit a left-right patterning defect, in which hindgut looping is predominantly sinistral (Hozumi et al., 2006). *ds* mutant flies had a mis-looped phenotype (Fig. 8B,I), which is thought to be due to mis-regulation of MyoID, which the Ds-ICD can physically associate with (González-Morales et al., 2015).

Looping of the hindgut can be visualized using ingestion of food with blue dye. Control *ds^Cr^*^+*:V5*^ flies all exhibited normal dextral looping (Fig. 8A,I). Deletion of the A motif did not result in significant hindgut looping defects (Fig. 8D,I). Conversely, complete ICD deletion, or deletion of just the D motif, resulted in a completely penetrant mis-looped phenotype like that of *ds* mutants (Fig. 8C,E,I). The smaller D-III deletion mutant also exhibited a completely penetrant mis-looped phenotype (Fig. 8H,I), but the D-II deletion exhibited a milder phenotype, with 19% of flies exhibiting normal dextral looping (Fig. 8G,I). The *ds^Cr^*^DCHS1*:V5*^ hybrid transgene also exhibited partial Ds activity in this assay, with normal dextral looping in 27% of flies (Fig. 8F,I).

MyoID binds to the Ds-ICD, but specific regions responsible for this binding have not been identified (González-Morales et al., 2015). To investigate this, we performed co-immunoprecipitation experiments on lysates of S2 cells expressing Flag-tagged MyoID and V5-tagged Ds-ICD (Fig. 8J). This established that deletion of the D motif eliminates MyoID binding, which is consistent with observations that the D motif disrupts normal hindgut looping. Among the smaller deletions, D-I did not reduce binding to MyoID, D-II reduced binding to 57% of control levels, although this was not statistically significant, and D-III reduced binding to 40% of control levels (Fig. 8K,L). The ability to associate with MyoID was conserved in the DCHS1 ICD (Fig. 8M). Like Dachs, MyoID is an atypical myosin, so its association with overlapping regions of the ICD suggests that this association could be mediated in part through aspects of myosin structure common to both proteins.

## DISCUSSION

We have examined the functional requirements for different sequence motifs within the Ds-ICD and assessed the ability of some known Ds interactors to associate with these motifs, to define roles of the Ds-ICD and investigate the hypothesis that Ds mediates downstream signal transduction. Amino acid sequence comparisons identified several conserved regions, which we divided into six motifs, four conserved to vertebrates and two only conserved within insects. Evolutionary conservation between insects and vertebrates implies that the motifs are functionally essential, but multiple assays identified only one of the four motifs conserved in vertebrates, the D motif, as having major effects on Ds activity, whereas deletion of the B, E, or F motifs had either no effect or only minor effects. We also attempted to address the significance of sequence conservation by assaying the ability of the human DCHS1 ICD to provide Ds-ICD function, using a hybrid transgene encoding the extracellular domain and transmembrane domain of *Drosophila* Ds and the ICD of DCHS1. However, the hybrid protein appears to be mis-localized, suggesting that it may be mostly misfolded, which could explain its failure to provide significant Ds activity *in vivo*. Nonetheless, co-immunoprecipitation experiments confirmed that key partners of *Drosophila* Ds, including Lft, Dachs, MyoID and Sple, could associate with the DCHS1 ICD in cultured cells.

The two motifs that had greatest effects on Ds activity, the A and D motifs, each affected Ds protein levels or membrane localization, and, consequently, a significant part of their effect could be due to effects on the levels of Ds at its normal location. Thus, we cannot attribute specific roles for these motifs in downstream signaling, and indeed their effects might be primarily due to increased or decreased activation of Fat by Ds. Importantly, deletion of the smaller D-II motif impaired Ds function without noticeably decreasing Ds levels. The C motif also had mild effects on Ds activity, both positive and negative, but it did not visibly affect binding to the partners we examined, so the basis for its requirement remains to be determined. The lack of other identifiable deletions impairing Ds function is somewhat surprising if Ds functions as a signal transducing receptor, but it could be that some functions are provided in a redundant fashion, such that larger deletions would be needed to significantly impair function. Alternatively, it could be that Ds functions primarily as ligand, with the principal function of the ICD being to modulate the levels and localization of the extracellular domain so that it can appropriately regulate Fat. We also note that as motifs that our molecular studies indicate should be crucial for PCP, i.e. interaction with Dachs and Sple, are embedded within a region that is also crucial for regulation of Ds membrane levels, we could not fully separate potential PCP activities downstream of Ds from regulation of PCP via Fat.

Our studies provide a molecular explanation for part of the impact of the D motif deletion on Ds by the discovery that this motif is necessary and sufficient for mediating physical association with Lft. Moreover, deletion of the D motif renders Ds insensitive to altered Lft expression. Lft was previously identified as being required for normal levels and distribution of Fat and Ds (Mao et al., 2009). Intriguingly, there is a region of sequence similarity between the Fat- and Ds-ICDs that partially overlaps with the Ds D region, which thus likely corresponds to a shared Lft-interacting sequence motif. This motif also has some similarity to sequences within the E-cadherin (Shotgun in *Drosophila*) ICD (Clark et al., 1995; Mao et al., 2009). However, the phenotype of animals with deletion of the Ds D region, or the smaller D-II and D-III regions, is more severe than the phenotype of *lft* mutants. This implies that the D region has other functions beyond mediating association with Lft.

One such function that we identified is interaction with Dachs, as co-immunoprecipitation experiments established that the D region is necessary and sufficient for association with Dachs. However, our studies also suggest that the ability of Ds to associate with Dachs may not be required for Dachs membrane localization or polarization, both of which are preserved in D-II deletion mutants, despite a significant reduction (74%) in Dachs binding. This supports the hypothesis that Dachs regulation is primarily mediated through the activity of the Fat ICD in antagonizing Dachs membrane localization rather than any ability of the Ds-ICD to promote Dachs membrane localization. A region of zebrafish Dchs1b that interacts with the microtubule regulator Ttc28 overlaps with parts of the D-II and D-III regions (Chen et al., 2018), so this could potentially be another factor affected by D-region deletions, as could ICD-mediated associations between Ds and Fat.

The D motif is also required for association of Ds with MyoID, which suggests that this region could function as a motif for interaction with unconventional myosins. This is intriguing as Dachs is not conserved in mammals, and we lack a good understanding of how Dchs1-Fat4 signaling regulates PCP in mammals. MyoID has been linked to left-right patterning in both vertebrates and invertebrates (Hozumi et al., 2006; Juan et al., 2018; Saydmohammed et al., 2018), and the observation that both Dachs and MyoID interact with the same, conserved region of the Ds-ICD supports the idea that myosin family proteins interacting with this motif might contribute to Dchs1-Fat4 modulation of PCP.

The mechanism by which the D-II region influences Ds function is not clear. The levels and localization of Ds appear like those of wild-type protein, and PCP is only mildly affected, but the increased wing size and elevated membrane Dachs levels indicate that Ds activity is compromised. As it is hard to understand how reduced Ds-Dachs binding would lead directly to elevated Dachs levels, we favor the hypothesis that this allele is defective in activating Fat. Molecular mechanisms associated with Fat activation by Ds have not been well defined. That is, activation of Fat involves binding by Ds, but whether Ds activates Fat by triggering Fat multimerization, conformational changes in Fat, recruitment of other proteins, or other mechanisms remains uncertain. The *ds^Cr^*^Δ*D-II:V5*^ allele may thus prove useful for future investigations of this question.

## MATERIALS AND METHODS

### *Drosophila* genetics

Fly crosses were performed at 25°C unless otherwise noted. The following previously described fly stocks were used: *w^1118^*, *ds^UA071^* (Adler et al., 1998), *ds^36D^* (Rodriguez, 2004), *Df(2L)ED87/SM6a* (RRID:BDSC_8677), *UAS-FLAG:lft, UAS-RNAi-lft* (Mao et al., 2009), *hh-GAL4* (*FBti0017278*), *Dachs:GFP* (Bosveld et al., 2012), *arm-lacZ* (FBti0023290), and *Act>stop>EGFP:Dachs* (Brittle et al., 2012). *ds*^Δ*Ex12*^, *ds^Cr+:V5^*, *ds^Cr+^*, *ds^Cr^*^Δ*A:V5*^, *ds^Cr^*^Δ*B:V5*^, *ds^Cr^*^Δ*C:V5*^, *ds^Cr^*^Δ*D:V5*^, *ds^CrD^*^Δ*II:V5*^, *ds^CrD^*^Δ*III:V5*^, *ds^Cr^*^Δ*E:V5*^, *ds^Cr^*^Δ*F:V5*^, *ds^Cr^*^Δ*ICD:V5*^, and *ds^CrDCHS1:V5^* were generated in this study. The *ds*^Δ*Ex12*^ allele was generated by CRISPR/Cas9-mediated genome editing and homology-dependent repair, using services of WellGenetics. Two guide RNAs (CRISPR Target Site 01: AGTTCAGTAGCTGAAAAGGATGG; CRISPR Target Site 02: ACACGGATGTAATCGAGCACTGG) were used to delete *ds* exon 12 and replaced with an RMCE cassette containing an attP cassette and a 3xP3-RFP selection marker. The RMCE cassette within *ds* was subsequently substituted by site-specific recombination either with wild-type *ds* sequences or with *ds* sequences in which one of the conserved motifs was deleted, or the entire Ds-ICD was deleted. We also generated additional fly lines in which the entire Ds-ICD was substituted with the ICD from human DCHS1 using microinjection services from BestGene. *ds* alleles were recombined with FRT40A for mitotic recombination. Mutant clones were generated at 72-84 h after egg laying (AEL) using *hs-FLP*, induced for 1 h at 37°C, and analyzed 48 h later*.* To make Flp-out Dachs:GFP clones, animals were cultured at 18°C for 7 days, heat-shocked for 5 min at 35°C, and then wing discs were dissected out 18-24 h later.

### Analysis of adult phenotypes

Adult male wings were dissected and mounted in Gary's Magic Mount (4:1 Canada Balsam:Methyl Salicylate). Wings from at least 15 flies were imaged using a Zeiss Axioplan2 microscope and a Progress camera. Digital images of wings were traced manually to measure area and roundness using Fiji (Schindelin et al., 2012)*.* Values were normalized to the average in controls and plotted using GraphPad Prism. Cross-vein spacing was calculated by tracing the length of vein L4 between cross-veins, divided by the length of vein L3. For wings with incomplete cross-veins, we approximated the crossing points on the L4 vein using the incomplete cross-vein. Hair polarity was scored in the anterior proximal region of the wing based on the angle of deviation from the normal axis and categorized as normal (<30°), weak (30°-90°), or strong (>90°) if 10% or more wing hairs showed a deviation. Adult wing hair polarity was also manually quantified in Fiji by measuring the polarity vector relative to a reference vector, focusing on the anterior proximal region of the wing between the L1 and L2 vein. For each wing, 35-70 wing hairs were examined and five or six wings were analyzed per genotype. To determine the angle, polarity vectors were drawn using Fiji's line tool, extending from the base of the wing hair to its tip. Wing hair polarity angles were plotted as a Rose plot using MATLAB (MathWorks). PCV loss was scored manually and categorized as follows: no phenotype, incomplete PCV loss, and complete PCV loss. To examine hindgut looping, newly eclosed flies (both male and female) were fed fly food containing 2.5% erioglaucine (Sigma-Aldrich, 861146), and the hindgut was examined through the cuticle (González-Morales et al., 2015).

### Immunostaining wing imaginal discs

Wing discs were dissected from third instar larvae at 96-120 h AEL, fixed for 15 min in 4% paraformaldehyde and washed and stained as described previously (Rauskolb and Irvine, 2019). Images were collected on a Leica SP8 confocal microscope. For comparison of expression levels amongst different genotypes, consistent magnification, resolution, laser power, and detector settings were used. Primary antibodies were used for staining were: mouse anti-V5 (Invitrogen, R960-25; preabsorbed at 1:10 dilution, then used at 1:50), rat anti-E-cadherin (Developmental Studies Hybridoma Bank, DCAD2-c; 1:200), chicken anti-β-gal (Abcam, ab9361; 1:100), rat anti-Fat (1:1500) (Feng and Irvine, 2009), rat anti-Ds (Yang et al., 2002), mouse anti-Wg (Developmental Studies Hybridoma Bank, 9A4; 1:300), rabbit anti-Dcr2 (Abcam, ab4732; 1:1600), rabbit anti-FLAG (Sigma-Aldrich, F3165; 1:200). Secondary antibodies were from Jackson ImmunoResearch, Invitrogen and Biotium (20137). DNA was labeled with Hoechst 33342 (Life Technologies).

### Quantification of Dachs polarity

Dachs polarity was calculated in Fiji by measuring the polarity vector relative to a reference vector pointing distally. For each image, seven to nine confocal sections were projected for analysis. To determine the Dachs polarity angle, polarity vectors were drawn using Fiji's line tool, extending from the center of the cell to the strongest Dachs accumulation. When a broad region of similar intensity was observed, vectors were directed toward the center of the highest Dachs accumulation. Polarity angles were plotted into a Rose plot using MATLAB.

### Sequence analysis

Clustal Omega (https://www.ebi.ac.uk/jdispatcher/msa/clustalo) was used to identify sequence conservation among Dachsous ICDs from different species (Madeira et al., 2022), which was then displayed using Jalview (https://www.jalview.org) (Waterhouse et al., 2009). Dachsous-ICD structure was predicted using AlphaFold (https://alphafold.ebi.ac.uk) (Jumper et al., 2021).

### Cell culture, immunoprecipitation and western blotting

S2 cells (DGRC) were cultured in Schneider's *Drosophila* Medium (Gibco, 21720001), supplemented with 10% fetal bovine serum (ATCC, 30-2020) and antibiotics (Gibco, 15240062), at 28°C. Cells were transfected using Effectene (QIAGEN, 301427), incubated for 44-48 h at 28°C. Plasmids used to transfect S2 cells included: Aw-GAL4, pUAST-FLAG:Lft, pUAST-FLAG:LIX1, pUAST-FLAG:LIX1L (Mao et al., 2009), pUAST-Dachs-:FLAG (Mao et al., 2006), pUAST-GFP:V5, pUAST-Ds-TM-ICD:V5 (Mao et al., 2009), pUAST-Sple-N:FLAG (Ambegaonkar and Irvine, 2015). FLAG-tagged MyoID was generated for this study by cloning cDNA clone SD01662 from *Drosophila* Genomics Research Center (DGRC) into a pUAST vector using NEBuilder HiFi DNA Assembly Master Mix (NEB, E2621L). Fat-ICD with a C-terminal Alfa-tag (Götzke et al., 2019) was cloned into pUAST for this study. Plasmids encoding different Ds-ICD motif deletion constructs with a C-terminal V5-tag were created in this study: pUAST-Ds-TM-ICD-ΔA:V5, pUAST-Ds-TM-ICD-ΔB:V5, pUAST-Ds-TM-ICD-ΔC:V5, pUAST-Ds-TM-ICD-ΔD:V5, pUAST-Ds-TM-ICD-ΔE:V5, pUAST-Ds-TM-ICD-ΔF:V5, pUAST-Ds-TM-ICD-ΔD-I:V5, pUAST-Ds-TM-ICD-ΔD-II:V5, pUAST-Ds-TM-ICD-ΔD-III:V5, pUAST-Ds-TM-DCHS1-ICD:V5 and pUAST-Ds- D+EGFP:V5. pUAST-Ds-TM-ICD:V5 (Mao et al., 2009) was used to PCR amplify the Fat signal peptide along with the part of the Ds-TM region by using the following primer set: Fwd Primer: GAACTCTGAATAGGGAATTGGGATGGAGAGGCTACTGCTCC; Rev Primer: ATGGATGAATAGAAAGATTCC. Plasmids containing ds-Exon12 with different motifs deletion were provided by WellGenetics. These plasmids were used as a template to amplify Ds-ICD sequence with different conserved motif deletions by using the following primer set: Fwd Primer: GGCAATTGGTCTACTGGTAGC; Rev Primer: GGAAATGTGGGGACACGGATGGGCGGAGGCGGATCCGGAAAACCCATCCCAAACCCCCTCTTGGGTTTGGACAGCACTCGTACCGGTCATCATCACCATCACCATTAAAATTCGTTAACAGATCTGCG. The above two sets of PCR products were gel purified and cloned in a pUAST-attB vector digested with EcoRI using NEBuilder HiFi DNA Assembly Master Mix (NEB, E2621L). Transfected cells were lysed in RIPA buffer (140 mM NaCl, 10 mM Tris-HCl pH 8.0, 1 mM EDTA pH 8.0, 1.0% Triton X-100, 0.1% SDS, and 0.1% sodium deoxycholate) supplemented with Protease Inhibitor Cocktail COMPLETE EDTA-Free (Roche, 11873580001) and Phosphatase Inhibitor Cocktail Set II (Millipore, 524625) for 30 min at 4°C. Cell debris was precipitated by centrifugation at 21,130 ***g*** for 20 min at 4°C, and 25 μl of cell lysate was saved for input samples. Lysates were then pre-cleared using 25 μl of Pierce Protein A agarose beads (Thermo Fisher Scientific, 20333). ChromoTek V5-Trap Magnetic Agarose beads (Proteintech, v5tma) and ChromoTek DYKDDDDK Fab-Trap™ Agarose (Proteintech, ffa) were used for immunoprecipitation. Precleared lysates were incubated with 25 μl of either V5 or Fab-Trap beads for 1 h. After binding beads were washed with dilution buffer (10 mM Tris-Cl pH 7.5, 150 mM NaCl, 0.5 mM EDTA pH 8.0) three times (15 min each). Protein samples were resolved on SDS PAGE gels (Bio-Rad, 456-1086) along with Precision Plus Dual Color Protein Standard (Bio-Rad, 1610394) and transferred to a nitrocellulose membrane (Bio-Rad, 170-4270) using a Trans-Blot Turbo machine (Bio-Rad). Membrane was blocked with Intercept Blocking Buffer (LI-COR, 927-70001) for 1 h at room temperature and then incubated with primary antibodies mouse anti-V5 (Invitrogen, R960-25; 1:5000), rabbit anti-FLAG (Sigma-Aldrich, F3165; 1:2000), FluoTag-X2 anti-ALFA antibody (1:2500; NanoTag Biotechnologies, N1502-Li800-L) and mouse anti-GAPDH (1:20,000; Proteintech, 60004-1-IG) overnight at 4°C. Blots were washed four times (10 min each) with PBT (1×PBS+0.01% Tween-20), then incubated with secondary antibodies anti-mouse IgG-800 (LI-COR Biosciences; 1:20,000) and anti-rabbit IgG-680 (LI-COR Biosciences; 1:20,000) for 1 h at room temperature. Blots were rinsed with PBT four times (10 min each) and then imaged using a LI-COR Odyssey CLX machine. For analysis of Ds in wing discs, discs from third-instar larvae were dissected in Ringer's Solution supplemented with Protease Inhibitor Cocktail and Phosphatase Inhibitor Cocktail (as above) on ice and then lysed in 2× Laemmli Sample Buffer (Bio-Rad, 1610737EDU) and heat denatured at 95*°*C for 5 min.

### Statistical analysis

Statistical analysis was performed using GraphPad Prism 10. Statistical tests for graphs showing wing area, roundness, and relative cross-vein distance were determined by one-way ANOVA. All quantifications for adult wing images are presented as mean±s.d. Quantifications for western blots are presented as mean±s.e.m. For all statistical tests, *P*-values are represented as *P≤0.05, **P≤0.01, ***P≤0.001, ****P≤0.0001.

## Supplementary Material



10.1242/develop.202919_sup1 Supplementary information
