# Supplementary material for: Contributions of the Dachsous intracellular domain to Dachsous-Fat signaling
Source: Development. 2024 Nov 29;151(23):dev202919. doi: 10.1242/dev.202919 (PMC11634027; doi:10.1242/dev.202919)
Supplement: Supplementary information [file develop-151-202919-s1.pdf]

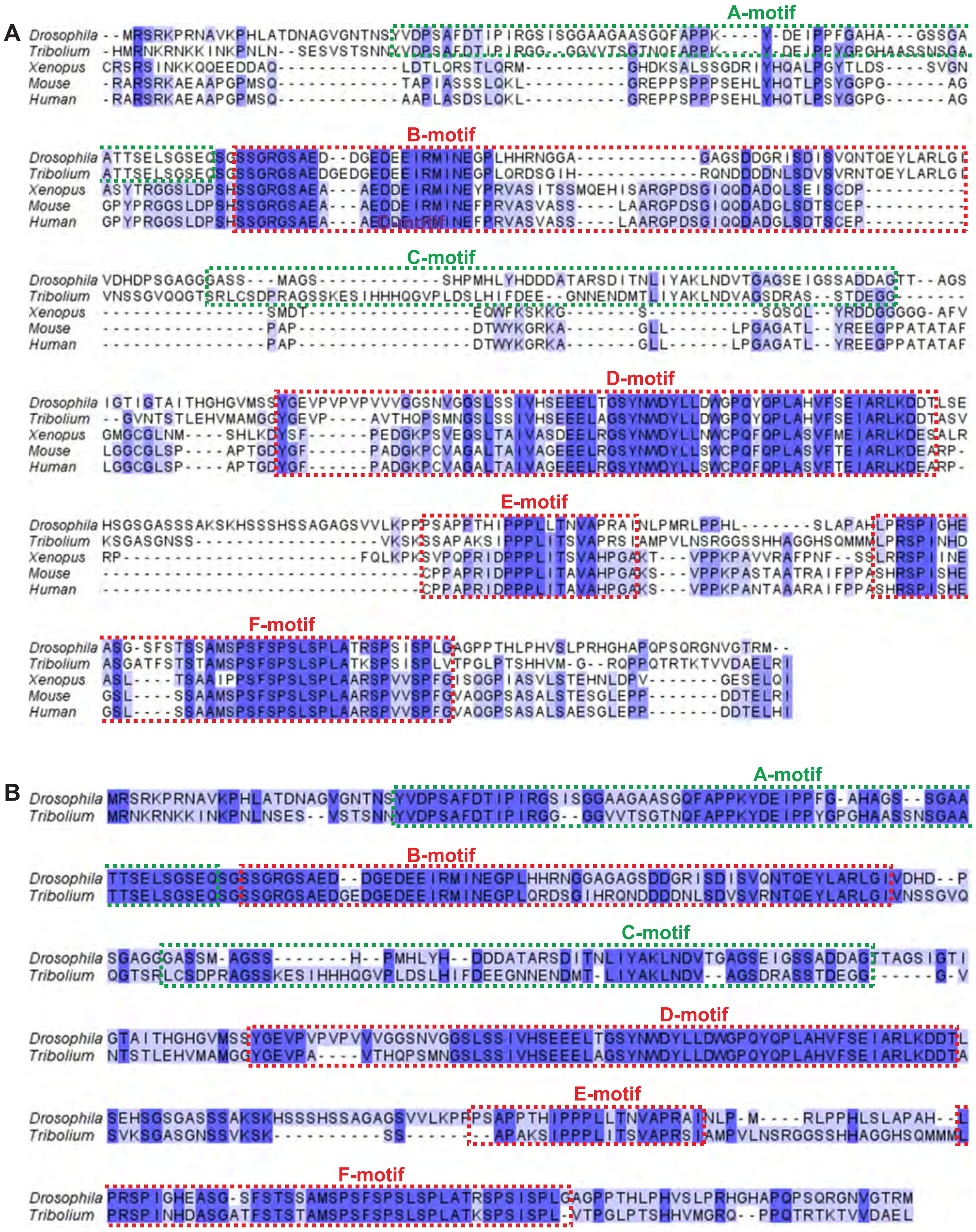
A
A-motif
B-motif
C-motif
D-motif
E-motif
F-motif
Drosophila
Tribolium
Xenopus
Mouse
Human
B
A-motif
B-motif
C-motif
D-motif
E-motif
F-motif
Drosophila
Tribolium

Fig. S1. *Dachsous* ICD amino acid sequence alignment

(A) Alignment of Dachsous ICD sequences from *Drosophila* (*Drosophila melanogaster*, NP_001285551.1, amino acids 3121-3556), *Tribolium* (*Tribolium castaneum*, KYB28093.1, amino acids 2398-2824), *Xenopus* (*Xenopus laevis*, XP_018105995.1, amino acids 2894-3239) Mouse (*Mus musculus*, NP_001156415.1, amino acids 2955-3291) and Human (*Homo sapiens*, NP_003728.1, amino acids 2962-3298). Amino acids identical among all five proteins are highlighted in dark blue color, and amino acids identical between three to four proteins are highlighted in lighter blue colors. **(B)** Alignment of Dachsous ICD sequences between *Drosophila* and *Tribolium*. For both (A) and (B), the conserved sequence motifs analyzed are outlined by dashed red boxes for sequences conserved from flies to humans and dashed green boxes for sequences conserved only within insects.

Development • Supplementary information

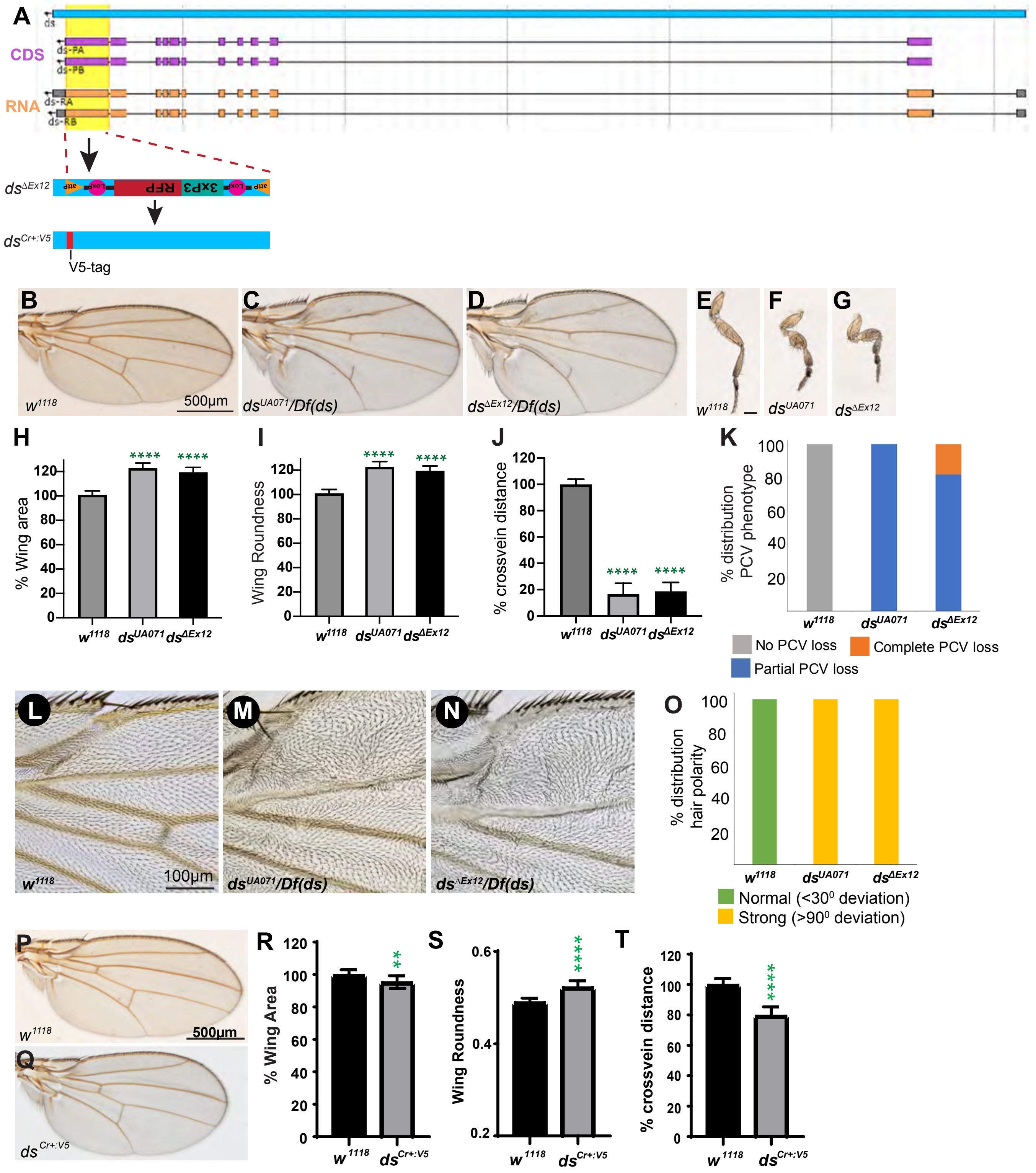

Development • Supplementary information

Fig. S2. Generation and characterization of a *ds* exon 12 deletion allele

(A) Map of the *ds* transcription unit (blue), CDS (purple), and exons (gray/orange) obtained from FlyBase JBrowse (Gramates et al., 2022; Jenkins et al., 2022). Exon 12 is highlighted in yellow, and structure of *ds* alleles is below. **(B-D)** Adult male wings from homozygous w^{1118} (B), hemizygous ds^{UA071}/ *Df(ds)* (C), hemizygous $ds^{\Delta Ex12}$/ *Df(ds)* (D). Scale bar = 500µm. **(E-G)** Adult male prothoracic legs from homozygous w^{1118} (E), hemizygous ds^{UA071}/ *Df(ds)* (F), hemizygous $ds^{\Delta Ex12}$/ *Df(ds)* (G). Scale bar = 200µm. **(H-J)** Histograms displaying relative wing area, wing roundness, and relative crossvein distance from the indicated genotypes. Each bar indicates mean ± SD, measurements from 25 wings for w^{1118}, 25 wings for ds^{UA071}/*Df(ds)*, and 11 wings for $ds^{\Delta Ex12}$/*Df(ds)*. **(K)** Histogram representing the comparison of the distribution of PCV loss phenotypes for these same genotypes. **(L-N)** Proximal anterior region of adult male wings from homozygous w^{1118} (L), hemizygous ds^{UA071}/ *Df(ds)* (M), hemizygous $ds^{\Delta Ex12}$/ *Df(ds)* (N). **(O)** Histogram showing the distribution of wing hair PCP phenotypes from the proximal wing for animals of the indicated genotypes, from 11-25 wings as described above. **(P,Q)** Adult male wings from homozygous w^{1118} (P), homozygous $ds^{Cr+:V5}$ (Q). Scale bar = 500µm. **R-T)** Histograms displaying % wing area, wing roundness, and relative crossvein distance from the indicated genotypes. Each bar indicates mean ± SD, measurements from 26 wings for w^{1118}, and 16 wings for $ds^{Cr+:V5}$.

Development • Supplementary information

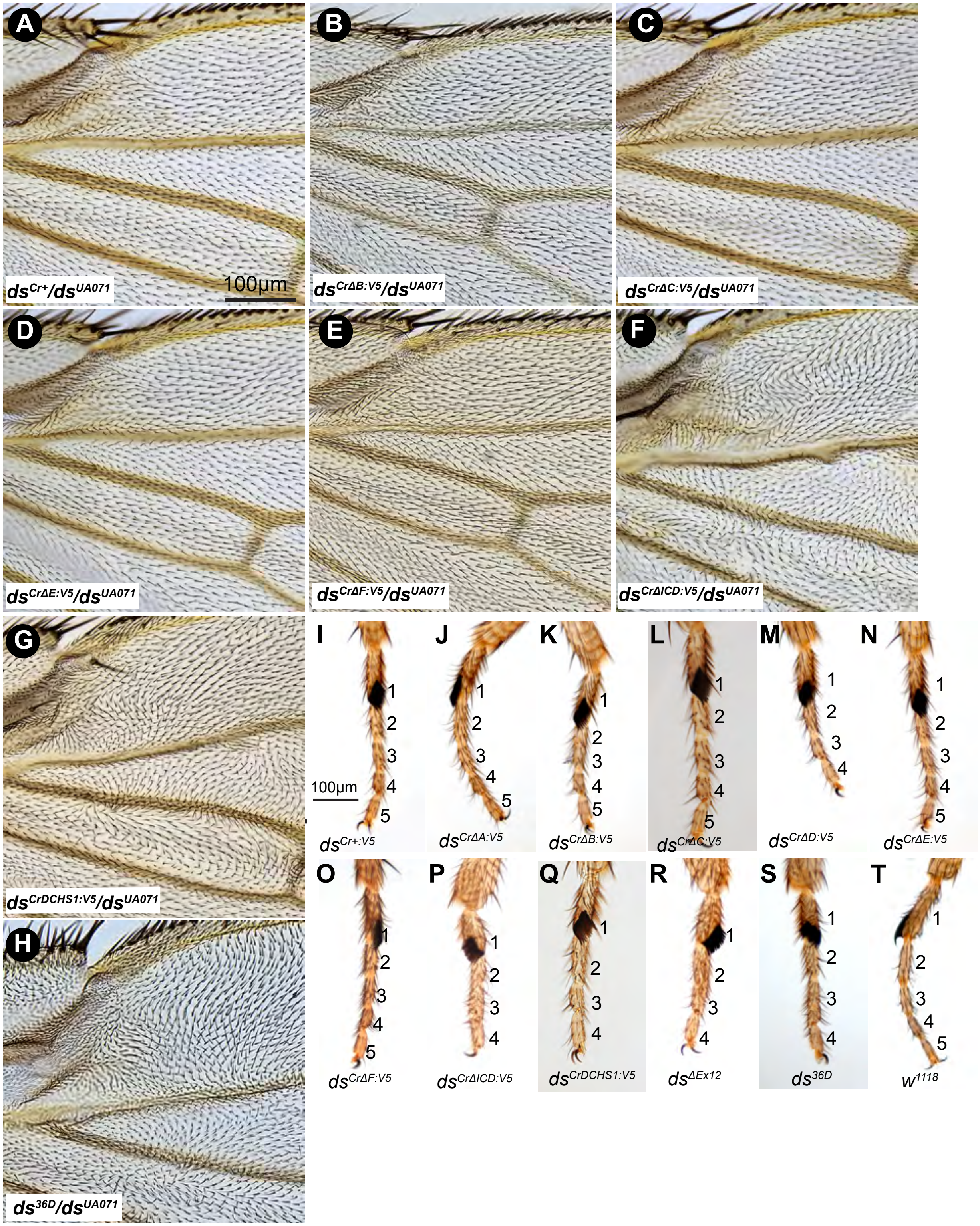
A
ds^{Cr+}/ds^{UA071}
100µm
B
$ds^{Cr\Delta B:V5}/ds^{UA071}$
C
$ds^{Cr\Delta C:V5}/ds^{UA071}$
D
$ds^{Cr\Delta E:V5}/ds^{UA071}$
E
$ds^{Cr\Delta F:V5}/ds^{UA071}$
F
$ds^{Cr\Delta ICD:V5}/ds^{UA071}$
G
$ds^{CrDCHS1:V5}/ds^{UA071}$
H
ds^{36D}/ds^{UA071}
I
100µm
$ds^{Cr+:V5}$
J
$ds^{Cr\Delta A:V5}$
K
$ds^{Cr\Delta B:V5}$
L
$ds^{Cr\Delta C:V5}$
M
$ds^{Cr\Delta D:V5}$
N
$ds^{Cr\Delta E:V5}$
O
$ds^{Cr\Delta F:V5}$
P
$ds^{Cr\Delta ICD:V5}$
Q
$ds^{CrDCHS1:V5}$
R
$ds^{\Delta Ex12}$
S
ds^{36D}
T
w^{1118}

Fig. S3. Wing hair PCP and tarsal leg phenotypes in *ds*-ICD mutants

(A-H) Proximal anterior wing regions of adult males of genotypes ds^{Cr+}/ds^{UA071} (A), $ds^{Cr\Delta B:V5}/ds^{UA071}$ (B), $ds^{Cr\Delta C:V5}/ds^{UA071}$ (C), $ds^{Cr\Delta E:V5}/ds^{UA071}$ (D), $ds^{Cr\Delta F:V5}/ds^{UA071}$ (E), $ds^{Cr\Delta ICD:V5}/ds^{UA071}$ (F), $ds^{CrDCHS1:V5}/ds^{UA071}$ (G), and ds^{36D}/ds^{UA071} (H). Scale bar = 100 μm. **(I-T)** Tarsal region of prothoracic legs from adult males of genotypes $ds^{Cr+:V5}/ds^{UA071}$ (I), $ds^{Cr\Delta A:V5}/ds^{UA071}$ (J), $ds^{Cr\Delta B:V5}/ds^{UA071}$ (K), $ds^{Cr\Delta C:V5}/ds^{UA071}$ (L), $ds^{Cr\Delta D:V5}/ds^{UA071}$(M), $ds^{Cr\Delta E:V5}/ds^{UA071}$ (N), $ds^{Cr\Delta F:V5}/ds^{UA071}$ (O), $ds^{Cr\Delta ICD:V5}/ds^{UA071}$(P), $ds^{CrDCHS1:V5}/ds^{UA071}$(Q), $ds^{\Delta Ex12}/ds^{UA071}$(R), ds^{36D}/ ds^{UA071}(S), and w^{1118}/ ds^{UA071}(T). Scale bar = 100 µm.

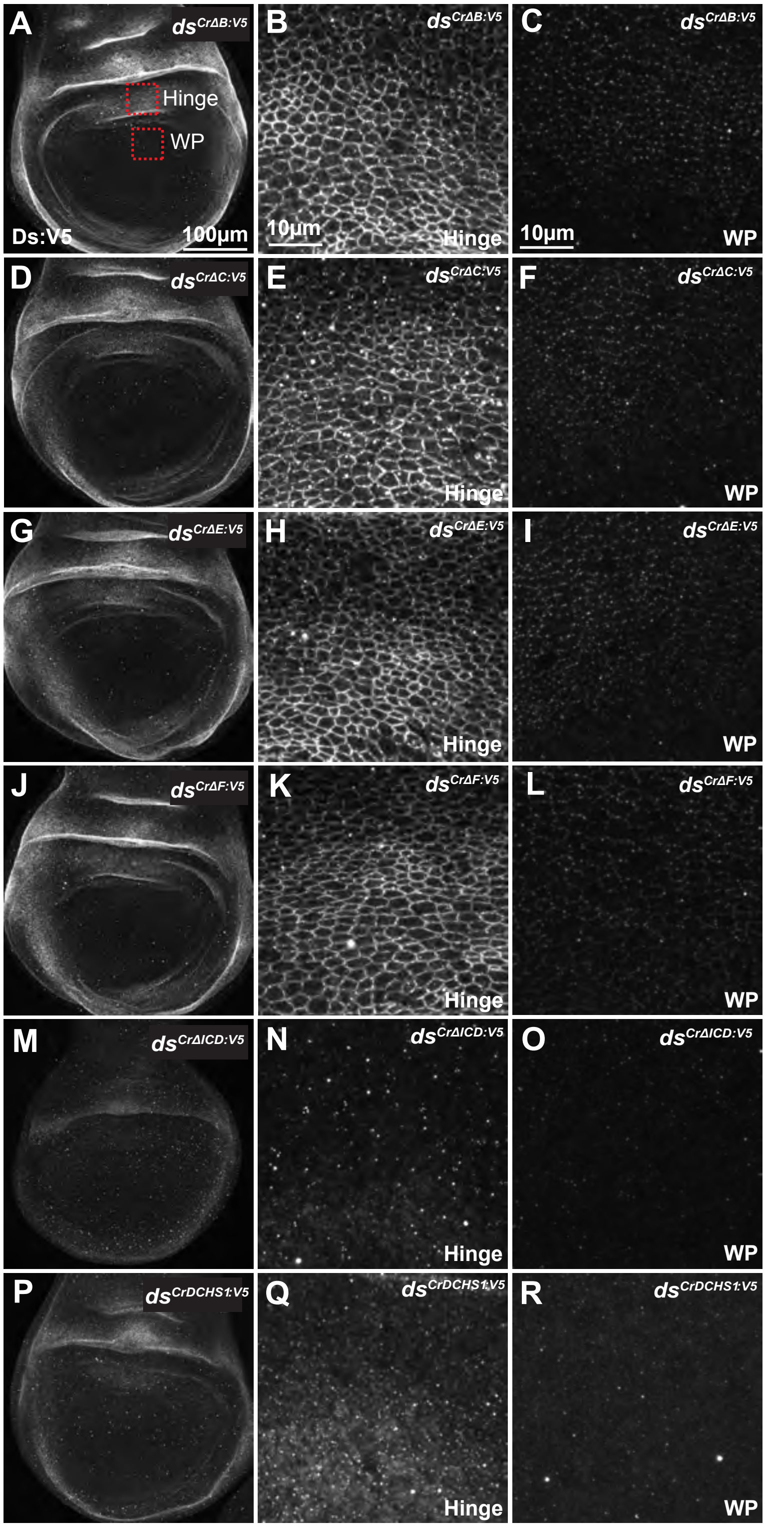
A
$ds^{Cr\Delta B:V5}$
Hinge
WP
Ds:V5
100µm
B
$ds^{Cr\Delta B:V5}$
10µm
Hinge
C
$ds^{Cr\Delta B:V5}$
10µm
WP
D
$ds^{Cr\Delta C:V5}$
E
$ds^{Cr\Delta C:V5}$
Hinge
F
$ds^{Cr\Delta C:V5}$
WP
G
$ds^{Cr\Delta E:V5}$
H
$ds^{Cr\Delta E:V5}$
Hinge
I
$ds^{Cr\Delta E:V5}$
WP
J
$ds^{Cr\Delta F:V5}$
K
$ds^{Cr\Delta F:V5}$
Hinge
L
$ds^{Cr\Delta F:V5}$
WP
M
$ds^{Cr\Delta ICD:V5}$
N
$ds^{Cr\Delta ICD:V5}$
Hinge
O
$ds^{Cr\Delta ICD:V5}$
WP
P
$ds^{CrDCHS1:V5}$
Q
$ds^{CrDCHS1:V5}$
Hinge
R
$ds^{CrDCHS1:V5}$
WP

Fig. S4. Localization of Ds:V5 proteins expressed by Ds-ICD deletions

Third-instar wing discs expressing Ds:V5 from homozygous $ds^{Cr\Delta B:V5}$ **(A-C)**, homozygous $ds^{Cr\Delta C:V5}$ **(D-F)**, homozygous $ds^{Cr\Delta E:V5}$ **(G-I)**, homozygous $ds^{Cr\Delta F:V5}$ (**J-L**), homozygous $ds^{Cr\Delta ICD:V5}$ (**M-O**), and homozygous $ds^{CrDCHS1:V5}$ (**P-R**). Red square boxes in (A) show the approximate locations in the hinge and wing pouch (WP) regions corresponding to the higher magnification panels at right. (**A,D,G,J,M,P**) Lower magnification images showing the entire wing pouch and hinge regions of the wing disc, Scale bar = 100 µm. **(B,E,H,K,N,Q)** Images depicting part of the wing hinge region in discs expressing the indicated V5-tagged Ds constructs. Scale bar = 10 µm. **(C,F,I,L,O,R)** Images depicting part of the proximal wing pouch in discs expressing V5-tagged Ds from the indicated V5-tagged Ds constructs.

Development • Supplementary information

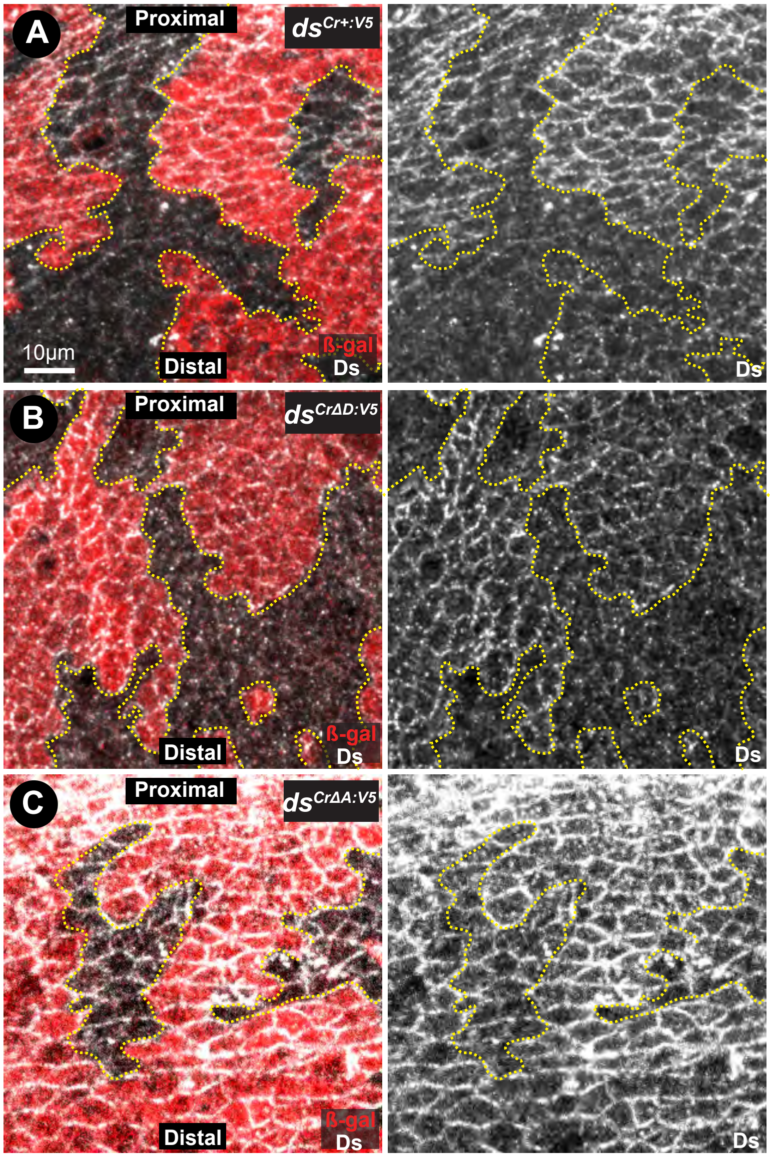

Fig. S5. Effect of ds^{Cr} alleles on Ds levels

Wing discs stained with Ds-antibody from *hs-Flp*; $ds^{Cr+:V5}$ *FRT40A/arm-lacZ FRT40A; Dachs:GFP/+* (A), *hs-Flp*; $ds^{Cr\Delta D:V5}$ *FRT40A/arm-lacZ FRT40A; Dachs:GFP/+* (B), or *hs-Flp*; $ds^{Cr\Delta A:V5}$ *FRT40A/arm-lacZ FRT40A; Dachs:GFP/+* (C), with mitotic clones homozygous for $ds^{Cr+:V5}$, $ds^{Cr\Delta D:V5}$, or $ds^{Cr\Delta A:V5}$, marked by loss of ß-gal (red), outlined with yellow dashes, to show effects on Ds levels (white). Scale bar = 10 µm.

Development • Supplementary information

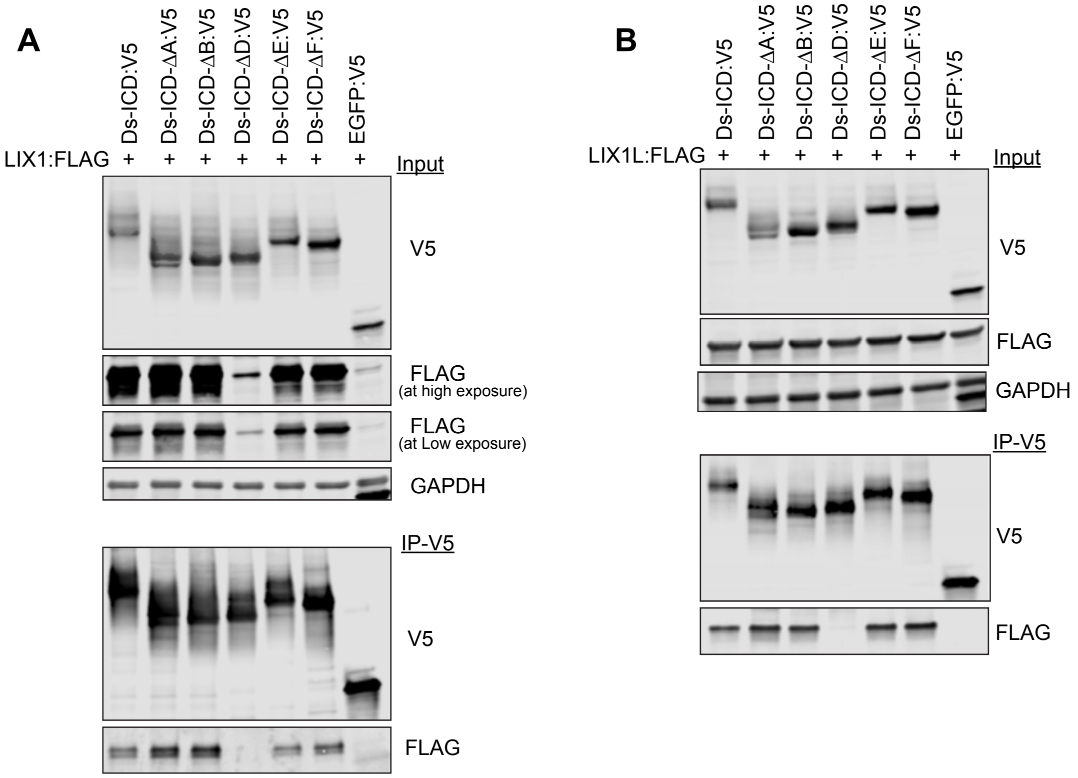

Fig. S6. The Ds-D motif associates with human LIX1 and LIX1-L

Western blots showing results of co-immunoprecipitation experiments between V5-tagged Ds ICD constructs and FLAG-tagged LIX1 **(A)** or LIX1-L **(B)** expressed in S2 cells. The top three panels show western blots on cell lysates expressing the indicated proteins, using the antibodies indicated at right. GAPDH is a control for loading and transfer. The bottom two panels show western blots on proteins immunoprecipitated with anti-V5 beads and detected with V5 or FLAG antibodies. V5-tagged EGFP is a negative control.

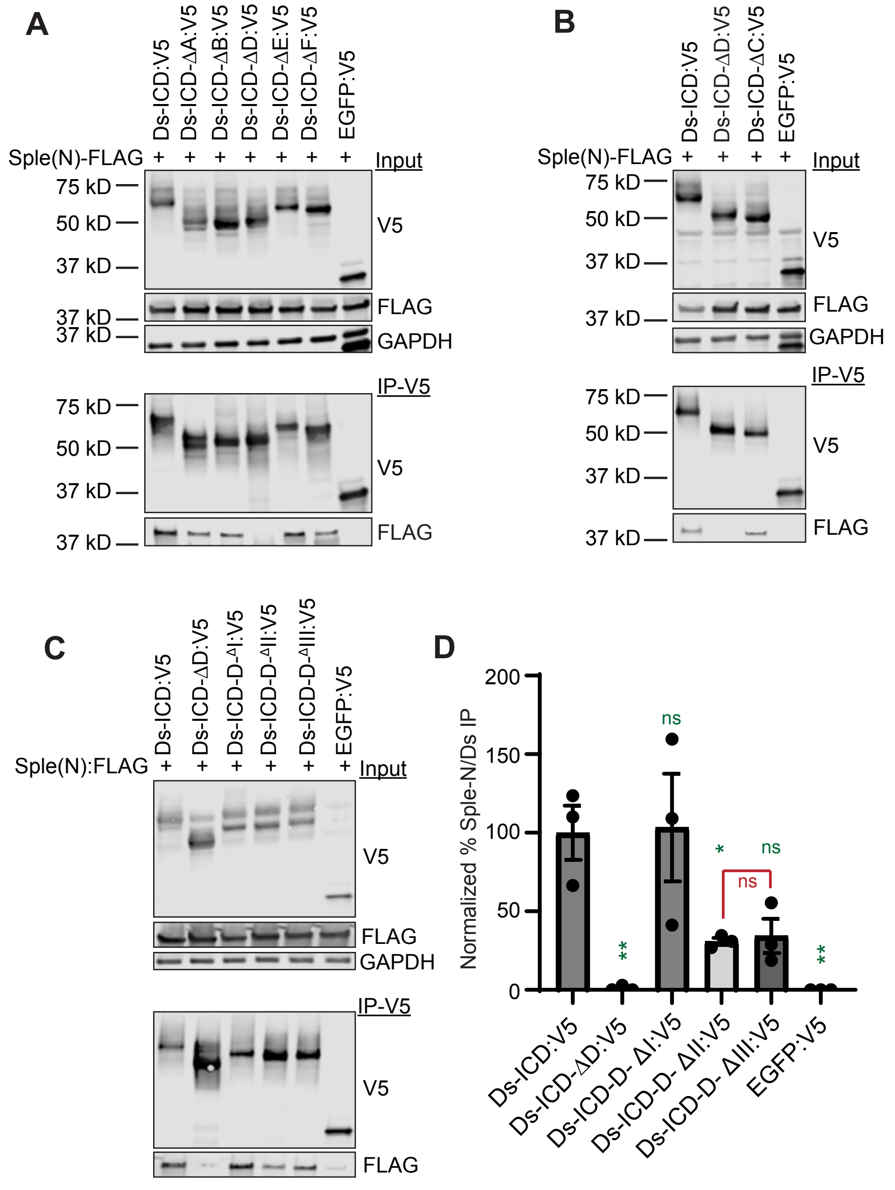

Fig. S7. Ds-D motif is required for association with Sple

(A-C) Western blots showing results of the co-immunoprecipitation experiments between V5-tagged Ds ICD constructs and FLAG-tagged Sple-N expressed in S2 cells. The top three panels show Western blots on cell lysates expressing the indicated proteins, using the antibodies indicated at right. GAPDH is a control for loading and transfer. The bottom two panels show western blots on proteins immunoprecipitated with anti-V5 beads and detected with V5 or FLAG antibodies. V5-tagged EGFP is a negative control. **(D)** Histogram showing quantification from 3 replicates of relative Sple-N association with different Ds ICD constructs, as illustrated in the example western blot in (C).

Development • Supplementary information

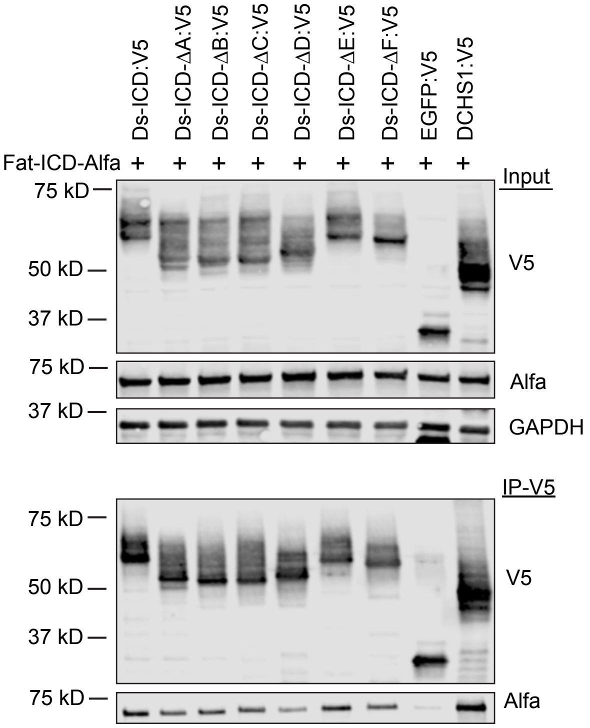

Fig. S8. Ds-D motif is required for association with Fat-ICD

Western blots showing results of the co-immunoprecipitation experiment between V5-tagged Ds ICD constructs and Alfa-tagged Fat-ICD expressed in S2 cells. The top three panels show Western blots on cell lysates expressing the indicated proteins, using the antibodies indicated at right. GAPDH is a control for loading and transfer. The bottom two panels show western blots on proteins immunoprecipitated with anti-V5 beads and detected with V5 or Alfa antibodies. V5-tagged EGFP is a negative control.

Development • Supplementary information

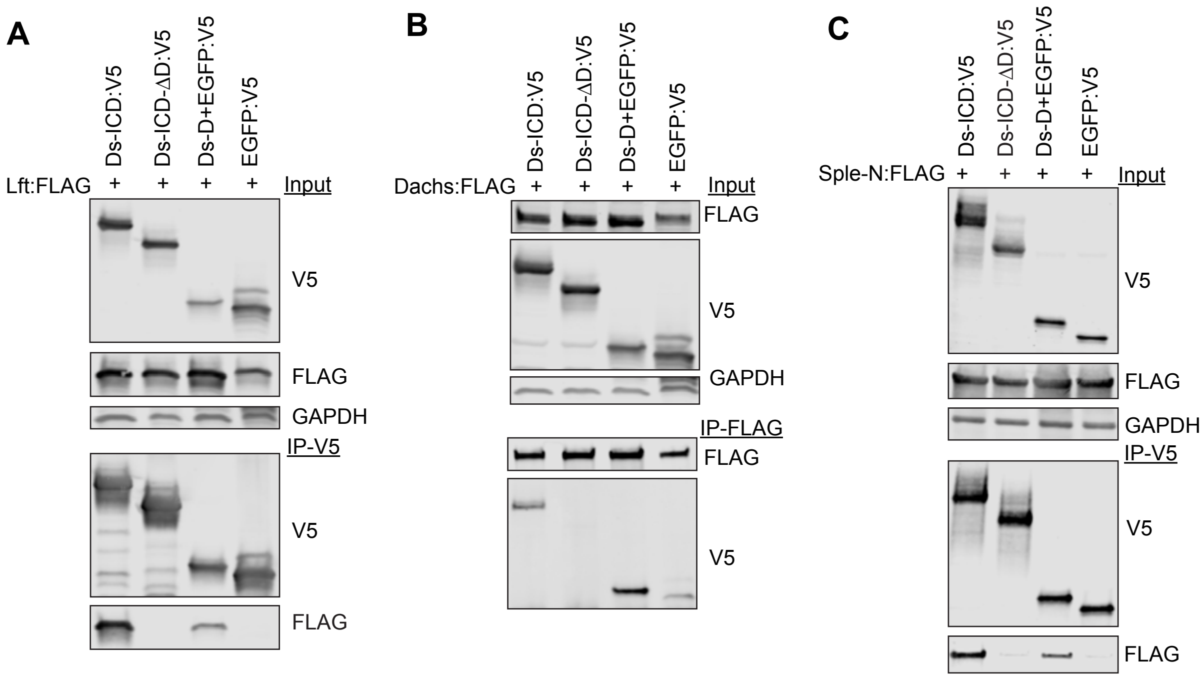

Fig. S9. The D region is sufficient for Lft, Dachs and Sple-N association

Western blot showing results of co-immunoprecipitation experiments between the indicated V5-tagged Ds constructs and FLAG-tagged Lft (A), Dachs (B), or Sple-N (C). Top three panels show blots on cell lysates expressing the indicated proteins, using antibodies indicated at right. Bottom two panels show blots on proteins immunoprecipitated with anti-V5 or Anti-FLAG beads and detected with V5 or FLAG antibodies.

Development • Supplementary information

Table S1. Summary of ds ICD deletion allele phenotypes. The effects of deletion of the indicated alleles across a range of phenotypes are summarized.

**Genotype**	**Wing Area**	**Wing Roundness**	**Crossvein distance**	**PCV loss phenotype**	**Hair Polarity**
$ds^{Cr\Delta A:V5}$	Reduced	Reduced	Increased	None	Normal
$ds^{Cr\Delta B:V5}$	No effect	No effect	No effect	Minimal	Normal
$ds^{Cr\Delta C:V5}$	Slightly increased	Slightly reduced	Slightly increased	Minimal	Normal
$ds^{Cr\Delta D:V5}$	Increased	Increased	Reduced	Strong	Mispolarized
$ds^{Cr\Delta E:V5}$	No effect	No effect	No effect	None	Normal
$ds^{Cr\Delta F:V5}$	No effect	No effect	No effect	None	Normal
$ds^{Cr\Delta ICD:V5}$	Increased	Increased	Reduced	Strong	Mispolarized
$ds^{CrDCHS1:V5}$	Increased	Increased	Reduced	Strong	Mispolarized

References

Gramates, L. S., Agapite, J., Attrill, H., Calvi, B. R., Crosby, M. A., Dos Santos, G., Goodman, J. L., Goutte-Gattat, D., Jenkins, V. K., Kaufman, T. et al. (2022). FlyBase: a guided tour of highlighted features. *Genetics* **220**, iyac035. doi:10.1093/genetics/iyac035

Jenkins, V. K., Larkin, A., Thurmond, J. and Flybase, C. (2022). Using FlyBase: a database of Drosophila genes and genetics. *Methods Mol. Biol.* **2540**, 1-34. doi:10.1007/978-1-0716-2541-5_1

Development • Supplementary information
