# Supplementary material for: Contributions of the Dachsous intracellular domain to Dachsous-Fat signaling
Source: Development. 2024 Nov 29;151(23):dev202919. doi: 10.1242/dev.202919 (PMC11634027; doi:10.1242/dev.202919)
Supplement: Supplementary file 1 [file develop-151-202919_review_history.pdf]

Contributions of the Dachsous intracellular domain to Dachsous-Fat signaling

Bipin Kumar Tripathi and Kenneth D Irvine
DOI: 10.1242/dev.202919

Editor: Thomas Lecuit

Review timeline

Original submission:	7 April 2024
Editorial decision:	28 May 2024
First revision received:	31 August 2024
Accepted:	9 October 2024

Original submission

First decision letter

MS ID#: DEVELOP/2024/202919

MS TITLE: Contributions of the Dachsous intracellular domain to Dachsous-Fat signaling

AUTHORS: Bipin Kumar Tripathi and Kenneth D Irvine

I have now received all the referees' reports on the above manuscript, and have reached a decision. The referees' comments are appended below, or you can access them online: please go to BenchPress and click on the 'Manuscripts with Decisions' queue in the Author Area.

As you will see, the referees express overall interest in your work, but two of them have some significant criticisms and recommend a substantial revision of your manuscript before we can consider publication. If you are able to revise the manuscript along the lines suggested, which does involve further experiments, I will be happy receive a revised version of the manuscript. Your revised paper will be re-reviewed by one or more of the original referees, and acceptance of your manuscript will depend on your addressing satisfactorily the reviewers' major concerns. Please also note that Development will normally permit only one round of major revision. If it would be helpful, you are welcome to contact us to discuss your revision in greater detail. Please send us a point-by-point response indicating your plans for addressing the referees' comments, and we will look over this and provide further guidance.

Please attend to all of the reviewers' comments and ensure that you clearly highlight all changes made in the revised manuscript. Please avoid using 'Tracked changes' in Word files as these are lost in PDF conversion. I should be grateful if you would also provide a point-by-point response detailing how you have dealt with the points raised by the reviewers in the 'Response to Reviewers' box. If you do not agree with any of their criticisms or suggestions please explain clearly why this is so.

Reviewer 1

Advance summary and potential significance to field

This manuscript conducts a structure-function analysis of the intracellular domain of Ds, in order to determine its contribution to Ft-Ds signaling. They make a series of deletions of conserved regions of the ICD and identify region A that enhances Ds levels and activity and region D that lowers Ds

© 2024. Published by The Company of Biologists under the terms of the Creative Commons Attribution License (https://creativecommons.org/licenses/by/4.0/).

levels and activity. They further go on to show that region D binds to Lowfat, Dachs, Spiny-legs and MyoID, and to partially sub-divide the functions of the D region.

The analysis is for the most part thorough and convincing. However the importance of some of the observed interactions is unclear. In particular, Lowfat is known to regulate Ds (and Ft) levels, and loss of Lft binding could explain most of the phenotypes observed. In contrast binding of Dachs to Ds does not seem to be necessary for Dachs membrane localization. It also remains unclear whether the PCP phenotypes are caused by a reduction in Ds levels (thus affecting signaling via Ft) or loss of Sple binding (particularly as Sple is not normally active in the wing).

Comments for the author

Minor comments:-

(1) The data described in Figures 1 and 2 is quite dense. It would be useful to have a summary table to show clearly which phenotypes are affected by which deletions, and a summary sentence or two to describe the overall conclusions.

(2) In Figure 1, please indicate in the legend the statistical test, and to which sample that the comparisons are made (dsCr+:V5, I think?).

(3) Also in Figure 1, the M&M say the statistics were determined by Paired t-tests. This is not paired data so I don't think this can be correct, and also an ANOVA should be used for multiple comparisons.

(4) In Figure S3, is panel Q a different magnification?

(5) In Figure 3, could you indicate in the figure legend what the Ds-150 and Ds-210 bands in the western blots are, and were both quantitated?

(6) How was Dachs polarity quantified in Figure 5? The legend also says how many clones were quantified but how many individual wing discs were used?

(7) For the AlphaFold Model in Figure 6, can the authors indicate the N and C-terminus, and also provide a version with the motifs D-I, D-II and D-III colored the same as in panel B (perhaps with D-I in a unique color)?

(8) In Figure 6, the authors conclude that motif D-II is preferentially required over motif D-III for association with Dachs, based on quantitation of IPs - but are the samples statistically different?

(9) I am confused by the dextral looping data in Figure 8. The text and images say that the ΔA mutant does not result in significant looping defects, but the graph appears to show only 90% are mis-looped, unless I have misunderstood. ΔD-II has 19% with normal dextral looping (i.e. 81% abnormal), yet the images shows a normal gut rather than the more common abnormal gut (and similarly for the DCHS1 version).

(10) In Figure 8, the authors say that ΔD-II has reduced binding to MyoID, while ΔD-I and ΔD-II do not reduce binding. However, I don't think this conclusion can be justified as this experiment only has an n of 2.

Reviewer 2

Advance summary and potential significance to field

The conserved protocadherin Dachsous (Ds) regulates growth, patterning, and planar cell polarization (PCP). Through its extracellular domain, Ds interacts with the Fat protocadherin and with Dachs, Lowfat, Spiny-legs (Sple), and MyoID via its intracellular domain. In this manuscript, the authors used genomic deletion to assess the roles of conserved domains of the Ds intracellular

© 2024. Published by The Company of Biologists under the terms of the Creative Commons Attribution License (https://creativecommons.org/licenses/by/4.0/).

region in wing growth, PCP, patterning, and gut looping. They found that a Ds domain (A domain) is necessary to decrease its membrane localization, while domain D is necessary to increase it. The D domain is shown to interact with the four known Ds interactors. By further dissection of the Ds D domain, the authors state that they have identified subdomains that preferentially bind to Lowfat and Dachs. Yet, this proposition needs to be better supported to sustain the authors' conclusions.

Comments for the author

The following points need to be addressed to improve the manuscript.
Major Points:
Point 1: The ds[Cr+] allele does not perform as a wild-type allele. The authors do not fully explore why this allele is not wild type. Its use as a baseline is reasonable. However, the authors do not provide an explanation for the observed partial loss of function, which somehow weakens the analysis and the manuscript findings. In addition, genomic methods can be deployed to create clean deletions in the wild-type background to test the function of each Ds domain more rigorously. The authors need to report the comparison of the phenotype of homozygote ds[Cr+] with wild-type controls. Overall, the abstract statement, "Genomic deletions of conserved sequences within Dachsous identified regions of the intracellular domain required for normal development," sounds too strong.
To evaluate the impact of each deletion in relation to the wild-type control, it will be important that the authors plot the wild-type control in each graph of the manuscript.
Could the authors stain ds[Cr+] and wild-type files with Ds antibody to assess whether the Ds[Cr+] levels are lower than endogenous Ds?
In Figure 2, the PCP phenotypes are only assessed qualitatively. Since the rescue allele fails to fully rescue the loss of function, it will be important to provide a quantitative assessment of PCP in each condition.
The deletion of the C domain affects Ds function relative to ds[Cr+]. The authors do not delve into the role of this region. While outside of the scope of this manuscript, could the authors comment on the possibility that the C domain affects the Ds interaction with its different binding partners?

Point 2: The authors report the role of each domain in controlling the level of junctional Ds. Ds has been found to be planar polarized. Therefore, it will be critical to assess whether Ds deletion constructs are correctly planar polarized and whether its lack of polarization in each deletion construct could correlate with the ones of Dachs or Sple.

Point 3: The authors tested whether overexpression of Lowfat modulates the level of ds[CrDeltaD:V5] to investigate whether the interaction between LowFat and the D domain is necessary to promote regulation of Ds level by LowFat. To further substantiate their conclusion, it will be relevant that the authors compare the localization of ds[CrDeltaD:V5] in control and Lowfat mutant conditions. This will test whether the D domain is the only domain necessary for Lowfat-dependent regulation of Ds level.

Point 4: The authors state: "Most clones in dsCr+:V5 wing discs exhibit normal, distally-oriented Dachs polarity (Figure 5D, G). Dachs is also normally polarized in dsCrDA:V5 wing discs (Figure 5E, G). Conversely, most Dachs:GFP clones in dsCrDD:V5 mutant wing discs have mispolarized Dachs (Figure 5F, G)." These statements are too imprecise. The authors need to rigorously and quantitatively analyze the impact of the different constructs on Dachs polarization. In addition, the authors state: "Deletion of the A, B, C, E, or F motifs did not impair the ability to bind Dachs." This conclusion seems incorrect since the deletion of the E domain clearly reduces the Ds-Dachs interaction as shown in Fig. 5. The authors need to explore more rigorously the function of this domain or to further sustain their conclusion. In Figure 5, could the authors stain the Ds full-length and deletion clones to further establish that the A and D domains modulate Ds level.

Point 5: The authors state: "The observation that each of three different binding partners of Ds: Lft, Dachs, and Sple, all require the D motif for association with Ds raised concerns about the specificity of this requirement." To address this point, they analyzed in vitro if the D domain can bind to Lowfat, Dachs, and Sple. The bindings of Lowfat and Dachs to the Ds D domain are clear in Figure S7. However, the binding to Sple is very weak. Therefore, the authors need to revise their conclusion or downplay it.

© 2024. Published by The Company of Biologists under the terms of the Creative Commons Attribution License (https://creativecommons.org/licenses/by/4.0/).

Point 6: Could the authors confirm that the deletion of the D-I domain does not increase the binding of Ds to Sple as suggested by the quantification in S6D? They also need to comment on these results in the main text. Based on this finding, would it be relevant to describe the phenotype of the Delta-I deletion?

Point 7: The authors write: "Thus, we could partially separate the sequence motifs required for association of Lft and Dachs with Ds, as the D-II region is preferentially required for association with Dachs and the D-III region is absolutely required for association with Lft." This conclusion sounds incorrect since the D-II is not preferentially required for the association with Dachs since the deletions of the II and III domains affect Dachs binding to a similar extent. Furthermore, and as stated and shown in the figure, the deletion of the D-II strongly impairs binding to Lowfat.
Lastly, the WB shown in figure 5G shows a clear reduction of the binding of Dachs to Ds-ICD-D-Delta-I.

Point 8: The authors claim that domain III is critically required for the interaction with Lowfat. Yet, this domain is not required to regulate Ds level (Fig 3). They, therefore, should conclude that both domain II and III are necessary for LowFat activity.

Point 9: While the deletion of domain II mildly affects Dachs polarity, the deletion of domain III drastically affects it. This finding is at odds with the authors' conclusion that they could partially separate Ds activity based on binding of Dachs to the different deletion constructs.

Point 10: The authors state: "One such function that we identified is interaction with Dachs, as co-immunoprecipitation experiments established that the D region is necessary and sufficient for association with Dachs. However, our studies also suggest that the ability of Ds to associate with Dachs is not required for Dachs membrane localization or polarization, both of which are preserved in D-II deletion mutants, despite a significant reduction (74%) in Dachs binding." This conclusion is incorrect. The deletion of domain III also strongly affects the binding of Ds to D and the experiments shown in Figure 7 show that Dachs polarization is strongly affected.

Minor Points:
In WB panels, the authors need to change Ds:V5 to Ds-ICD.
The panel I is missing in Figure 6.

<u>Reviewer 3</u>

Advance summary and potential significance to field

Overall, this a novel, well executed study that dissects the function of the intracellular region of the cell adhesion molecule Ds, both in vivo and biochemically. Regions of conservation are assessed for in vivo function through deletion analysis, in known functions of Ds in PCP and growth controls. Interestingly that the "D" region of Ds is most important for PCP and wing size. Loss of the D region blocks the ability of overexpressed Lowfat to stabilize Ds. Biochemical analyses show that the D region is required to CoIP Ds with Lft. Loss of the D region also leads to increased subapical Dachs levels, and leads to mispolarized Dachs (as shown in a nice mosaic analysis of Dachs-GFP) and loss of the D region blocked the ability of Ds to coIP with Dachs. Loss of the D region also blocked the ability of Ds to bind Sple.

Given the importance of the D region for PCP and growth, and the fact that the D region binds Sple, Dachs and Lowfat, they further dissect the D region into three subregions(D-I, D-II and D-III). Deleting the DIII region blocked binding of Lft, with a smaller contribution by DII. Similarly, Dachs is largely (but not fully) dependent on the DIII region, and less of the DII. Sple is only affected by 20% by deletion of DII or DIII.
Deletion of DII and DIII in vivo gave similar phenotype with DIII having the strongest effects. DIII also affects Ds localization to junctions. Dachs levels are increased with loss of DII and DIII, though the polarity effects are largely due to DIII (possibly separating the Dachs levels and polarity effects of Ds). DIII also phenocopies the looping effects of loss of Ds or of loss of the entire D region. Interestingly, their analysis of the binding of MyoID to Ds showed that DII was important for MyoD binding.

© 2024. Published by The Company of Biologists under the terms of the Creative Commons Attribution License (https://creativecommons.org/licenses/by/4.0/).

Comments for the author

My suggestions are largely textual, or re formatting of the figures to make them more clear for the reader.

1. Loss of Ds leads to some pupal lethality. What is the effect of homozygous loss of ICD on viability? This was not clear. In the discussion, address the lethality of the loss of Ds ICD- and if there is no lethality, why loss of the Ds ICD does not have as strong an effect as the null.

2. Not necessary, but if an antibody against Ds is available, it would be helpful to know what the actual changes in protein levels are in the lines with the "normal" Ds ICD replacement. This might help clarify why the ‘control’ (DsCr+ or DsCr+:V5) , which is very different from w1118, and address why the biggest difference in roundness and crossvein distance is between w1118 and all the others Ds deletion lines.

3. The cell shapes seem affected by loss of D, (3H). Is this panel representative? please comment if so.
Ds ICD has been shown to bind to the Ft ICD, this could be discussed in the context of the effects of the loss of the D region in interaction with Ft
Fig 3 WB: mention what DsII and DsIII are in legend – possibly refer forward to the section dealing with subdivisions.
General: legends in individual figure panels are very small and hard to read
The discussion would benefit from touch on the separation of the need to bind MyoID and phenotype for the D subdivision mutants.

First revision

Author response to reviewers' comments

Response to Reviewers

We sincerely thank all three reviewers for their valuable comments and suggestions, which have significantly improved our manuscript. Within the manuscript text, changes and additions are indicated by purple text.

Reviewer 1: Advance Summary and Potential Significance to Field:
This manuscript conducts a structure-function analysis of the intracellular domain of Ds, in order to determine its contribution to Ft-Ds signaling. They make a series of deletions of conserved regions of the ICD and identify region A that enhances Ds levels and activity and region D that lowers Ds levels and activity. They further go on to show that region D binds to Lowfat, Dachs, Spiny-legs and MyoID, and to partially sub-divide the functions of the D region.

The analysis is for the most part thorough and convincing. However, the importance of some of the observed interactions is unclear. In particular, Lowfat is known to regulate Ds (and Ft) levels, and loss of Lft binding could explain most of the phenotypes observed. In contrast binding of Dachs to Ds does not seem to be necessary for Dachs membrane localization. It also remains unclear whether the PCP phenotypes are caused by a reduction in Ds levels (thus affecting signaling via Ft) or loss of Sple binding (particularly as Sple is not normally active in the wing).

Reviewer 1 Comments for the
Author: Minor comments:-
(1) The data described in Figures 1 and 2 is quite dense. It would be useful to have a summary table to show clearly which phenotypes are affected by which deletions, and a summary sentence or two to describe the overall conclusions.

Response: As suggested, we have now created a table (Table 1) summarizing the effect of

© 2024. Published by The Company of Biologists under the terms of the Creative Commons Attribution License (https://creativecommons.org/licenses/by/4.0/).

different deletions on the phenotypes examined in Figs 1 & 2, and added a brief summary of conclusions at the end of the section describing these results.

(2) In Figure 1, please indicate in the legend the statistical test, and to which sample that the comparisons are made (dsCr+:V5, I think?).

Response: We have revised the Figure 1 legend to include this information (One-way ANOVA, and *ds*$^{Cr+:V5}$).

(3) Also in Figure 1, the M&M say the statistics were determined by Paired t-tests. This is not paired data, so, I don't think this can be correct, and also an ANOVA should be used for multiple comparisons.

Response: Agreed, one-way ANOVA is more appropriate here we have revised the figure and Methods accordingly.

(4) In Figure S3, is panel Q a different magnification?

Response: It was not a different magnification, although we agree it looked slightly larger. We have replaced it with a different example that is more representative.

(5) In Figure 3, could you indicate in the figure legend what the Ds-150 and Ds-210 bands in the western blots are, and were both quantitated?

Response: We have added text to the legend to better explain these bands, which result from alternative processing of Ds. Both are included in the quantitation of Ds protein levels.

(6) How was Dachs polarity quantified in Figure 5? The legend also says how many clones were quantified, but how many individual wing discs were used?

Response: Previously, Dachs polarity was manually scored in individual clones as either normal or mispolarized. In the revised manuscript, we have measured and plotted angles of polarization measuring a polarity vector in Fiji, as described in the revised Methods. We have also now added the number of individual wing discs analyzed to the figure legend.

(7) For the AlphaFold Model in Figure 6, can the authors indicate the N and C-terminus, and also provide a version with the motifs D-I, D-II and D-III colored the same as in panel B (perhaps with D-I in a unique color)?

Response: We have made the suggested modifications.

(8) In Figure 6, the authors conclude that motif D-II is preferentially required over motif D-III for association with Dachs, based on quantitation of IPs - but are the samples statistically different?

Response: We have now performed statistical tests on these data, which reveal that the difference is not significant. We have revised the text to reflect this.

(9) I am confused by the dextral looping data in Figure 8. The text and images say that the ΔA mutant does not result in significant looping defects, but the graph appears to show only 90% are mis-looped, unless I have misunderstood. ΔD-II has 19% with normal dextral looping (i.e. 81% abnormal), yet the images shows a normal gut rather than the more common abnormal gut (and similarly for the DCHS1 version).

Response: There was an error in the plotting of the ΔA mutant, in which around 96% of the flies have a normal dextral looped hindgut. We have corrected the graph. We have also changed the images displayed for DCHS1 and ΔD-II hindgut phenotypes to reflect the predominant, mis- looped, phenotype.

(10) In Figure 8, the authors say that ΔD-II has reduced binding to MyoID, while ΔD-I and ΔD-II do

© 2024. Published by The Company of Biologists under the terms of the Creative Commons Attribution License (https://creativecommons.org/licenses/by/4.0/).

not reduce binding. However, I don't think this conclusion can be justified as this experiment only has an n of 2.

Response: We now have performed additional experiments, and also performed statistical tests on these results. Our new data show that both ΔD-II and ΔD-III deletions reduce binding to MyoID, although the reduction with ΔD-II was not statistically significant. ΔD-I has no effect.

***** Reviewer 2 Advance Summary and Potential Significance to Field:
The conserved protocadherin Dachsous (Ds) regulates growth, patterning, and planar cell polarization (PCP). Through its extracellular domain, Ds interacts with the Fat protocadherin and with Dachs, Lowfat, Spiny-legs (Sple), and MyoID via its intracellular domain. In this manuscript, the authors used genomic deletion to assess the roles of conserved domains of the Ds intracellular region in wing growth, PCP, patterning, and gut looping. They found that a Ds domain (A domain) is necessary to decrease its membrane localization, while domain D is necessary to increase it. The D domain is shown to interact with the four known Ds interactors. By further dissection of the Ds D domain, the authors state that they have identified subdomains that preferentially bind to Lowfat and Dachs. Yet, this proposition needs to be better supported to sustain the authors' conclusions.

Reviewer 2 Comments for the Author:
The following points need to be addressed to improve the manuscript.

Major Points:
Point 1: The ds[Cr+] allele does not perform as a wild-type allele. The authors do not fully explore why this allele is not wild type. Its use as a baseline is reasonable. However, the authors do not provide an explanation for the observed partial loss of function, which somehow weakens the analysis and the manuscript findings. In addition, genomic methods can be deployed to create clean deletions in the wild-type background to test the function of each Ds domain more rigorously. The authors need to report the comparison of the phenotype of homozygote ds[Cr+] with wild-type controls. Overall, the abstract statement, "Genomic deletions of conserved sequences within Dachsous identified regions of the intracellular domain required for normal development," sounds too strong.
To evaluate the impact of each deletion in relation to the wild-type control, it will be important that the authors plot the wild-type control in each graph of the manuscript.
Could the authors stain ds[Cr+] and wild-type files with Ds antibody to assess whether the Ds[Cr+] levels are lower than endogenous Ds?

Response: Unfortunately the wild-type control alleles that we generated (*ds*$^{Cr+}$ and *ds*$^{Cr+:V5}$) have mild *ds* phenotypes, despite having no mutations in the *ds* coding region. We think this is due to small sequences inserted by the cassette exchange strategy we employed, which might affect expression by eg affecting transcript stability. As our goal is to assess the effects of deletions in the ICD on Ds function, we reasoned that we could still use the alleles we had generated and characterized, but use *ds*$^{Cr+}$ and *ds*$^{Cr+:V5}$ as points of comparison, rather than the parental wild-type stock (*w*^{1118}).
To address the issues raised by the reviewer here, in the revised manuscript, we added stains with Ds antibody on clones in wing discs, which confirm that the level of Ds protein expression in *ds*$^{Cr+:V5}$ cells is lower than in cells with a wild-type allele (new Fig S5), which can explain the mild phenotypes observed. We had included a wild-type (*w*^{1118}) control for all the comparisons in Fig. 1, and have now also added a comparison of *ds*$^{Cr+:V5}$ homozygotes to *w*^{1118} homozygotes to Fig. S2. The results are very similar those observed in transheterozygous combinations, as reported in Fig. 1. We also changed the phrase "identified regions of the intracellular domain required for normal development" to "identified regions of the intracellular domain that contribute to Ds function".

In Figure 2, the PCP phenotypes are only assessed qualitatively. Since the rescue allele fails to fully rescue the loss of function, it will be important to provide a quantitative assessment of PCP in each condition.

Response: We initially quantified polarity by classifying wings as having either normal polarity

© 2024. Published by The Company of Biologists under the terms of the Creative Commons Attribution License (https://creativecommons.org/licenses/by/4.0/).

(hairs deviate less than 30° from normal distal orientation), weak polarity phenotypes (30-90° deviation) or strong polarity phenotypes, with 14-30 wings analyzed per genotype. As hairs are mispolarized in different directions in different regions of the wing, and we were not able to effectively automate quantitation of hair polarity, to address the reviewers comment we chose a small anterior-proximal region to quantify the polarity of individual hairs within genotypes that disturb polarity. This was done by drawing lines along hairs to measure hair direction in Fiji. 5-6 wings were analyzed per genotype, with 35-70 wing hairs were examined per wing. Hair polarity directions were then plotted in a rose plot using MATLAB, and these new results added to Figs. 2 and 6.

The deletion of the C domain affects Ds function relative to ds[Cr+]. The authors do not delve into the role of this region. While outside of the scope of this manuscript, could the authors comment on the possibility that the C domain affects the Ds interaction with its different binding partners?

Response: We checked various binding partners (Lft, Dachs, Sple, Myo1D) but did not observe effects of C-motif deletion in co-IP assays. However, as the effect of C-motif deletion on phenotypes is subtle, it's possible that potential effects on interaction with binding partners were too subtle to be detected. Alternatively, it might act in some other way. We have now added a comment about the C motif to the discussion.

Point 2: The authors report the role of each domain in controlling the level of junctional Ds. Ds has been found to be planar polarized. Therefore, it will be critical to assess whether Ds deletion constructs are correctly planar polarized and whether its lack of polarization in each deletion construct could correlate with the ones of Dachs or Sple.

Response: We agree this would be interesting. However, the region that significantly impacts polarity (D-motif) also impacts Ds protein levels, and we found that when we tried to measure Ds polarity in $ds^{Cr\Delta D:V5}$ wing discs, the protein levels were too low for us to determine whether or how it is polarized.

Point 3: The authors tested whether overexpression of Lowfat modulates the level of ds[CrDeltaD:V5] to investigate whether the interaction between LowFat and the D domain is necessary to promote regulation of Ds level by LowFat. To further substantiate their conclusion, it will be relevant that the authors compare the localization of ds[CrDeltaD:V5] in control and Lowfat mutant conditions. This will test whether the D domain is the only domain necessary for Lowfat-dependent regulation of Ds level.

Response: To address this, we used RNAi-Lft to knockdown the Lft levels, which decreased staining of Ds:V5 from the control $ds^{Cr+:V5}$ allele, but did not further reduce the already low levels of Ds:V5 from the $ds^{Cr\Delta D:V5}$ allele. These new results have been added to Fig. 4.

Point 4: The authors state: "Most clones in dsCr+:V5 wing discs exhibit normal, distally-oriented Dachs polarity (Figure 5D, G). Dachs is also normally polarized in dsCrDA:V5 wing discs (Figure 5E, G). Conversely, most Dachs:GFP clones in dsCrDD:V5 mutant wing discs have mispolarized Dachs (Figure 5F, G)." These statements are too imprecise. The authors need to rigorously and quantitatively analyze the impact of the different constructs on Dachs polarization.

Response: We have now quantified Dachs polarity for these genotypes by measuring a polarity vector in Fiji, based on drawing a line from the center of the cell to the strongest Dachs accumulation, and plotting the resulting polarity vectors in Rose plots added to Figs 5 and 7.

In addition, the authors state: "Deletion of the A, B, C, E, or F motifs did not impair the ability to bind Dachs." This conclusion seems incorrect since the deletion of the E domain clearly reduces the Ds-Dachs interaction as shown in Fig. 5. The authors need to explore more rigorously the function of this domain or to further sustain their conclusion.

Response: We noticed that in the blot we displayed previously, the amount of Ds-ICDΔE:V5 input was low, which could explain why the band appeared lower in the co-IP blot. We performed

© 2024. Published by The Company of Biologists under the terms of the Creative Commons Attribution License (https://creativecommons.org/licenses/by/4.0/).

additional experiments and replaced the blot with another example, which we think is representative and does not show a reduced co-IP band.

In Figure 5, could the authors stain the Ds full-length and deletion clones to further establish that the A and D domains modulate Ds level.

Response: We have now performed the suggested experiments, using Ds-antibody, and observed that the levels of Ds protein staining are in higher in $ds^{Cr\Delta A:V5}$ and lower in $ds^{Cr\Delta D:V5}$ as compared to $ds^{Cr+:V5}$ clones. These new results are in a new Fig. S5.

Point 5: The authors state: "The observation that each of three different binding partners of Ds: Lft, Dachs, and Sple, all require the D motif for association with Ds raised concerns about the specificity of this requirement." To address this point, they analyzed in vitro if the D domain can bind to Lowfat, Dachs, and Sple. The bindings of Lowfat and Dachs to the Ds D domain are clear in Figure S7. However, the binding to Sple is very weak. Therefore, the authors need to revise their conclusion or downplay it.

Response: We noticed that in the blot we displayed previously, the amount of Ds-D+EGFP:V5 input was quite low, which could have caused the co-IP band to appear weak. We have now done additional co-IP experiments, and replaced the previous image with a different example where the binding to Sple is more clearly visible (Figure S9C).

Point 6: Could the authors confirm that the deletion of the D-I domain does not increase the binding of Ds to Sple as suggested by the quantification in S6D? They also need to comment on these results in the main text. Based on this finding, would it be relevant to describe the phenotype of the Delta-I deletion?

Response: The apparent increase was an artefact. We have performed additional co-IPs, and also now performed statistical tests, which show that the level of Sple binding to D-ΔI is not significantly different from the Ds-ICD control. These new results are in a revised Fig. S7.

Point 7: The authors write: "Thus, we could partially separate the sequence motifs required for association of Lft and Dachs with Ds, as the D-II region is preferentially required for association with Dachs and the D-III region is absolutely required for association with Lft." This conclusion sounds incorrect since the D-II is not preferentially required for the association with Dachs since the deletions of the II and III domains affect Dachs binding to a similar extent. Furthermore, and as stated and shown in the figure, the deletion of the D-II strongly impairs binding to Lowfat. Lastly, the WB shown in figure 5G shows a clear reduction of the binding of Dachs to Ds-ICD- D-Delta-I.

Response: Although there are some differences in the strength of binding detected to D-II and D-III region deletions, statistical tests that we have now performed indicate that these differences are not significant, so we have now modified the text to reflect this. Once we normalize for input amounts, Ds-ICD-D-ΔI does not show significantly reduced binding to Dachs; this is reflected in the quantitation shown in Fig. 6G.

Point 8: The authors claim that domain III is critically required for the interaction with Lowfat. Yet, this domain is not required to regulate Ds level (Fig 3). They, therefore, should conclude that both domain II and III are necessary for LowFat activity.

Response: We think Lowfat primarily affects the levels of Ds protein at junctions, rather than total protein levels, which is why the levels of Ds protein as measured by western blot are not significantly different from control for any of ΔD, DΔII, or DΔIII (Fig 3J,K), even though the levels appear reduced at cell junctions for by immunostaining ΔD and DΔIII (but not DΔII). This is why we infer that D-III is critically required for regulation by Lowfat.

Point 9: While the deletion of domain II mildly affects Dachs polarity, the deletion of domain III drastically affects it. This finding is at odds with the authors' conclusion that they could partially

© 2024. Published by The Company of Biologists under the terms of the Creative Commons Attribution License (https://creativecommons.org/licenses/by/4.0/).

separate Ds activity based on binding of Dachs to the different deletion constructs.

Response: We think that the influence of D-III deletion on Dachs polarity is mainly due to lower levels of Ds to the membrane, whereas for the D-II deletion the levels of Ds are similar to control, which could enable it to regulate Dachs through Fat. We revised the text to try to clarify this.

Point 10: The authors state: "One such function that we identified is interaction with Dachs, as coimmunoprecipitation experiments established that the D region is necessary and sufficient for association with Dachs. However, our studies also suggest that the ability of Ds to associate with Dachs is not required for Dachs membrane localization or polarization, both of which are preserved in D-II deletion mutants, despite a significant reduction (74%) in Dachs binding." This conclusion is incorrect. The deletion of domain III also strongly affects the binding of Ds to D and the experiments shown in Figure 7 show that Dachs polarization is strongly affected.

Response: As for our response to point 9, for comparison between these deletions we factor in the differences in levels of Ds on the membrane. We think that the influence of D-III deletion on Dachs polarity could be explained by lower levels of Ds at the membrane, whereas for the D-II deletion the levels of Ds are similar to control, which evidently enables it to regulate Dachs through Fat, despite the reduction in Dachs binding by the Ds ICD with this deletion. We revised the text to try to clarify this.

Minor Points:
In WB panels, the authors need to change Ds:V5 to Ds-ICD. The panel I is missing in Figure 6.

Response: We have changed all the labels for Ds:V5 to Ds-ICD:V5, as suggested. We're not sure why panel I wasn't visible for the reviewer's copy of Fig. 6, but we have made sure it's visible on the revised manuscript.

***** Reviewer 3 Advance Summary and Potential Significance to Field:
Overall, this a novel, well executed study that dissects the function of the intracellular region of the cell adhesion molecule Ds, both in vivo and biochemically. Regions of conservation are assessed for in vivo function through deletion analysis, in known functions of Ds in PCP and growth controls. Interestingly that the "D" region of Ds is most important for PCP and wing size. Loss of the D region blocks the ability of overexpressed Lowfat to stabilize Ds. Biochemical analyses show that the D region is required to CoIP Ds with Lft. Loss of the D region also leads to increased subapical Dachs levels, and leads to mispolarized Dachs (as shown in a nice mosaic analysis of Dachs-GFP) and loss of the D region blocked the ability of Ds to coIP with Dachs. Loss of the D region also blocked the ability of Ds to bind Sple.

Given the importance of the D region for PCP and growth, and the fact that the D region binds Sple, Dachs and Lowfat, they further dissect the D region into three subregions (D-I, D-II and D- III). Deleting the DIII region blocked binding of Lft, with a smaller contribution by DII. Similarly, Dachs is largely (but not fully) dependent on the DIII region, and less of the DII.
Sple is only affected by 20% by deletion of DII or DIII. Deletion of DII and DIII in vivo gave similar phenotype with DIII having the strongest effects. DIII also affects Ds localization to junctions. Dachs levels are increased with loss of DII and DIII, though the polarity effects are largely due to DIII (possibly separating the Dachs levels and polarity effects of Ds). DIII also phenocopies the looping effects of loss of Ds or of loss of the entire D region. Interestingly, their analysis of the binding of MyoID to Ds showed that DII was important for MyoD binding.

Reviewer 3 Comments for the Author:
My suggestions are largely textual, or re formatting of the figures to make them more clear for the reader.
1. Loss of Ds leads to some pupal lethality. What is the effect of homozygous loss of ICD on viability? This was not clear. In the discussion, address the lethality of the loss of Ds ICD- and if there is no lethality, why loss of the Ds ICD does not have as strong an effect as the null.

Response: As suggested, we have now examined the pupal lethality for the Ds-ICD deletion and

© 2024. Published by The Company of Biologists under the terms of the Creative Commons Attribution License (https://creativecommons.org/licenses/by/4.0/).

compared it with some other genotypes. For two control stocks (w^{1118}, and dsCr+:V5) we observed 97 and 98%, respectively, of pupae eclosed. For two strong *ds* allelic combinations (dsUA071/ds36D and dsΔEx12 homozygotes) we observed 51 and 45% of pupae eclosed, respectively. For dsCr-ICD:V5 homozygotes we observed we observed 68% of pupae eclosed. Thus, there does seem to be a modest improvement in viability (N=100-130 for all genotypes). The slight reduction in pupal lethality with the ICD deletion could mean that a small fraction of Ds protein with ICD deletion can be stable enough to bind Fat and affect downstream signaling. However, as this is not something we have investigated in detail we have not included it in the manuscript.

2. Not necessary, but if an antibody against Ds is available, it would be helpful to know what the actual changes in protein levels are in the lines with the "normal" Ds ICD replacement. This might help clarify why the ‘control’ (DsCr+ or DsCr+:V5) , which is very different from w1118, and address why the biggest difference in roundness and crossvein distance is between w1118 and all the others Ds deletion lines.

Response: We were able to use a small aliquot of Ds antibody to perform the suggested experiment, through immunostaining mitotic clones. This revealed that the Ds levels appear reduced in $ds^{Cr+:V5}$ cells compared to cells with wild-type *ds* (Figure S5). As the reviewer notes, the reduced level of Ds can explain the mild phenotype associated with our CRISPR control alleles ds^{Cr+} and $ds^{Cr+:V5}$.

3. The cell shapes seem affected by loss of D, (3H). Is this panel representative? please comment if so.

Response: Cell shapes are not affected by this allele, so we have replaced this panel with a different example.

Ds ICD has been shown to bind to the Ft ICD, this could be discussed in the context of the effects of the loss of the D region in interaction with Ft

Response: To address this, we have performed additional experiments examining binding between the Ds-ICD and Fat-ICD in co-IP experiments. Interestingly, deletion of the D-motif from Ds-ICD reduced the interaction of the Ds-ICD with the Fat-ICD. We added these new results to a new Figure S8.

Fig 3 WB: mention what DsII and DsIII are in legend – possibly refer forward to the section dealing with subdivisions.

Response: We revised the figure legend as suggested.

General: legends in individual figure panels are very small and hard to read

Response: We have made some changes to increase small font sizes in the figures.

The discussion would benefit from touch on the separation of the need to bind MyoID and phenotype for the D subdivision mutants.

Response: Additional analysis and quantitation show some reduction in MyoID binding for both the D-II and D-III deletions, which matches the observation that they both often have a mis- looped hindgut, although we note that the reduction for D-II was not statistically significant.

Second decision letter

MS ID#: DEVELOP/2024/202919

© 2024. Published by The Company of Biologists under the terms of the Creative Commons Attribution License (https://creativecommons.org/licenses/by/4.0/).

MS TITLE: Contributions of the Dachsous intracellular domain to Dachsous-Fat signaling

AUTHORS: Bipin Kumar Tripathi and Kenneth D Irvine
ARTICLE TYPE: Research Article

I apologise for the delay in getting back to you. I am happy to tell you that your manuscript has been accepted for publication in Development, pending our standard publication integrity checks.

Reviewer 1

Advance summary and potential significance to field

Very nice revisions. Strongly support publication.

Comments for the author

No further suggestions.

Reviewer 2

Advance summary and potential significance to field

The authors have thoroughly addressed my previous comments and improved their manuscript. I am pleased to recommend it for publication

Comments for the author

NA

Reviewer 3

Advance summary and potential significance to field

The authors have responded well to previous criticisms and have here provided novel insights into the role of conserved regions of the intracellular domain of Dachsous, using in vivo CRISPR, confocal and western blot analyses. This study highlights the importance of the conserved "D" region in Dachsous activity. The manuscript is now suitable for publication in Development.

Comments for the author

The manuscript has been substantially improved in the revised version.

© 2024. Published by The Company of Biologists under the terms of the Creative Commons Attribution License (https://creativecommons.org/licenses/by/4.0/).
